# Impact of structural coherence and disorder on the ionic transport and lattice dynamics in Li^+^-conducting argyrodites

**DOI:** 10.1039/d5ta07185b

**Published:** 2025-10-07

**Authors:** Thorben Böger, Kyra Strotmann, Vasiliki Faka, Oliver Maus, Douglas L. Abernathy, Garrett E. Granroth, Niina H. Jalarvo, Cheng Li, Emmanuelle Suard, Wolfgang G. Zeier

**Affiliations:** a Institute of Inorganic and Analytical Chemistry, University of Münster 48149 Münster Germany; b International Graduate School for Battery Chemistry, Characterization, Analysis, Recycling and Application (BACCARA), University of Münster 48149 Münster Germany; c Neutron Scattering Division, Oak Ridge National Laboratory Oak Ridge 37831 TN USA; d Diffraction Group, Institute Laue-Langevin (ILL) 71 Avenue des Martyrs 38000 Grenoble France; e Institute of Energy Materials and Devices (IMD), IMD-4: Helmholtz-Institut Münster: Ionics in Energy Storage Forschungszentrum Jülich 48149 Münster Germany wzeier@uni-muenster.de

## Abstract

Solid-state batteries offer improved safety and higher energy density compared to conventional lithium-ion systems. Among candidate solid electrolytes, lithium argyrodites stand out for their exceptional ionic conductivity and compositional flexibility. Recent studies have revealed strongly anharmonic, liquid-like ion and lattice dynamics in these materials, including the collapse of soft phonons driven by Li^+^ diffusion, which impacts both local vibrations and thermal transport. Yet, the connection between the local structure, phonon dynamics, and macroscopic heat transport remains unresolved. In this work, we employ post-synthesis processing to tune microstructural parameters—such as crystallite size, strain, and coherence length—in two model systems: Li_5.5_PS_4.5_Cl_1.5_ and Li_6_PS_5_Br. We systematically examine how mechanical treatments influence structural coherence, ion and lattice dynamics, and thermal transport. To further probe the role of structural disorder, we investigate bromide substitution in Li_6_PS_5_I. Across all compounds, thermal transport above 100 K is dominated by diffusons. At lower temperatures, however, structural disorder is significantly more effective than reduced coherence length at suppressing phonon-gas-type transport, underscoring the crucial role of the local structure. Together with a detailed analysis of lithium-ion dynamics, these results provide new insights into how structural coherence and disorder govern both transport and vibrational properties in fast ionic conductors.

## Abstract 2

While thermal conductivity is almost unaffected by post-synthesis treatments of lithium argyrodites, reducing coherence length and crystallite size, it is changed significantly by structural disordering suppressing phonon gas-type thermal transport.

## Graphical abstract

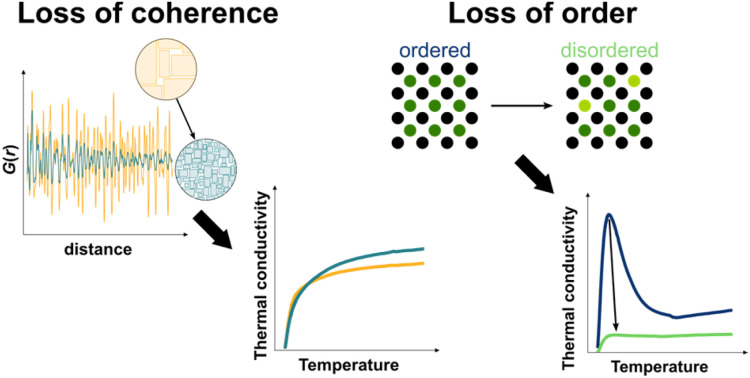

## Introduction

The ongoing electrification of processes that were previously reliant on fossil fuels has created a growing need for efficient electrical energy storage solutions. Electrochemical energy storage systems in the form of batteries are widely used today, but employ almost exclusively liquid electrolytes.^1^ In contrast, solid-state batteries utilize a solid electrolyte and promise several potential benefits over traditional lithium-ion batteries.^2^ These include enhanced safety and energy density due to the possibility of bipolar stacking^3^ and the use of advanced anodes such as silicon (alloy)^4^ or even lithium metal.^5,6^ A solid electrolyte suited for application in a solid-state battery should possess (kinetic) electrochemical stability against active materials, high ionic conductivity, and good processibility.^7^

**Fig. 1:**
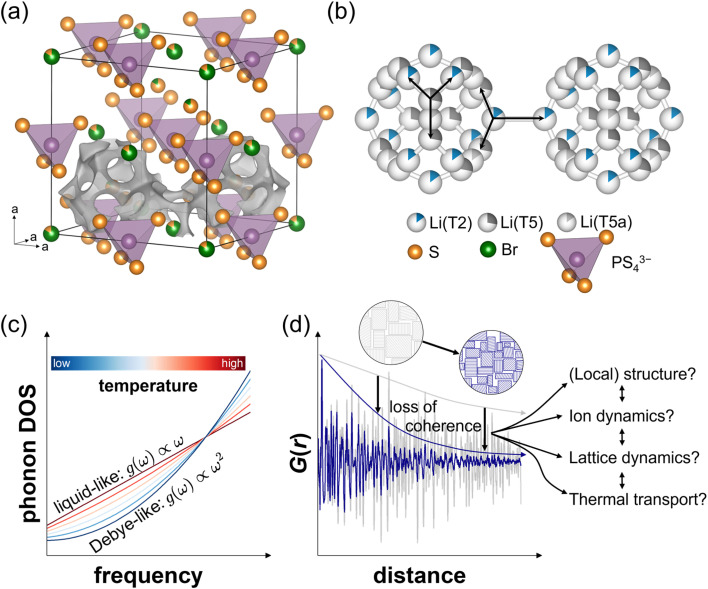
(a) The cubic polymorph of lithium argyrodites, featuring cages of Li^+^ ions surrounding a sulfide ion. Two of these cages are illustrated *via* a bond valence sum isosurface (gray). This bond valence sum isosurface represents the probability density of lithium ions at elevated temperatures, where the lithium ion sublattice exhibits highly dynamic, liquid-like behavior. (b) Interconnection of Li^+^ sites. Intra- and inter-cage jumps are visualized with black arrows. While a T5 site is connected to two T2 sites and another T5 site (*via* the intermediary T5a site), lithium ions on T2 sites can migrate to two adjacent T5 sites or *via* an inter-cage jump to another T2 site. (c) Schematic transition from Debye-like to liquid-like phonon DOS upon warming due to the increased mobility of Li^+^ ions. At elevated temperatures the diffusion of Li^+^ ions leads to a non-zero phonon DOS at a frequency of zero. (d) Mechanical input leads to a loss in coherence seen by a more pronounced decay of the reduced pair distribution function *G*(*r*) and schematically shown *via* exemplary microstructures. The loss of coherence, which is accompanied by smaller crystallite and particle sizes, may affect other quantities including structural properties, lattice and ion and thermal transport dynamics. This work aims to elucidate the effect of different processing procedures on such properties.

Among the many classes of solid electrolytes, the lithium argyrodite family stands out due to its high ionic conductivity and various possibilities for iso- and aliovalent substitutions.^8^ Although orthorhombic and monoclinic low temperature phases exist, the high temperature cubic structure (space group *F*4̄3*m*, Fig. 1a and b) exhibits far superior ionic conductivity, making it the focus of research. To stabilize the cubic polymorph at room temperature, halide ions are typically substituted into the argyrodite structure leading to a general formula of Li_7−*y*_PS_6−*y*_X_*y*_ (X = Cl, Br, I; 0 ≤ *y* ≤ 1.5). In this structure, the halide ions (Wyckoff 4a) form a face-centered cubic (fcc) lattice with PS_4_^3−^ polyanions occupying the octahedral voids, while “free” sulfide ions fill half of the tetrahedral voids (4d position). Both the chloride and bromide ions show anion site disorder with the free sulfide ions.^9^ The lithium ions occupy tetrahedrally coordinated sites forming face-sharing polyhedra that create partially occupied cages around the 4d position (Fig. 1a and b). The occupancy and distribution of lithium ions with respect to the halide ion,^9^ disorder,^10^ and amount of halides^11^ has been extensively studied in the literature. In short, in Li_5.5_PS_4.5_Cl_1.5_ the T2 and T5 positions (both 48h) are occupied, whereas in Li_6_PS_5_Br Li^+^ ions are also found to populate the trigonal-planar coordinated T5a positions (24g position) at the saddle point in between two T5 positions (Fig. 1b). Intra-cage diffusion of Li^+^ ions can occur *via* T5–T5a–T5 or T2–T5 jumps. For long-range transport, Li^+^ ions need to be able to jump from one cage to another. This inter-cage transport takes place *via* T2–T2 jumps.

Solely by halide substitution ionic conductivities of ≈10 mS cm^−1^ at room temperature have been achieved.^12,13^ The extent to which lattice vibrations influence ionic transport, and whether these vibrations can be systematically tuned to enhance ionic conductivity, is still an open question, not only in lithium argyrodites but also in solid electrolytes in general. By using a combination of quasi-elastic neutron scattering (QENS), inelastic neutron scattering (INS), and machine-learned molecular dynamics simulations, Ding *et al.*^14^ recently showed that upon heating, the phonon dynamics in Li_6_PS_5_Cl change. With increasing temperature and fast ion diffusion, the Li^+^ sublattice softens and heavily dampens the phonons, indicative of a potential energy surface with significant anharmonicity. Due to the high mobility of Li^+^ ions, vibrations associated with Li^+^ were found to break down entirely at elevated temperatures, leading to a linear phonon density of states (DOS) *g*(*ω*) ∝ *ω* at low frequencies *ω*, instead of the conventional quadratic Debye scaling law *g*(*ω*) ∝ *ω*^2^. This altered frequency dependency suggests a liquid-like ion dynamics (Fig. 1c) at high temperatures for low frequency vibrations. Additionally, by selectively restricting the degrees of freedom of specific parts of the host lattice, they reported host vibrations, especially of PS_4_^3−^ tetrahedra, to enhance the Li^+^ diffusivity by an order of magnitude over a frozen host lattice. This enhancement is attributed to the “dynamic breathing” of local bottlenecks along the diffusion pathways. It seems that by transitioning from Debye behavior of vibrations to a more liquid vibrational characteristic, the fast ionic conduction of lithium argyrodites needs to be understood by Li^+^ diffusion in a strong anharmonic regime, suggesting more focus on individual ion vibrations rather than average vibrational frequencies. To further explore the ion dynamics, phononic properties and ultimately thermal transport in lithium argyrodites, this work investigates how coherence length and local vibrations influence their structural and transport properties (Fig. 1d).

Measurements of ionic conductivity and electrolyte properties cannot be universally generalized for a given solid electrolyte composition, but are a function of the synthesis method,^15^ the particle size,^16,17^ and the structural coherence length.^18^ While particle size and coherence length are interrelated concepts, they describe phenomena on vastly different length scales. Particle size refers to the dimensions of secondary solid electrolyte agglomerates, typically in the micrometer range. In contrast, coherence length measures the distance over which atomic order is maintained. Strain and reduced crystallite size diminish the coherence length, and both require harsh mechanical treatment, which in turn also reduces particle size.^18,19^ Therefore, smaller coherence length and crystallite size are often accompanied by smaller particle size. The measurement of electrolyte properties as a function of their coherence length gives a measure for the importance of local factors in those properties. For example, Maus *et al.*^18^ investigated the influence of different post-synthesis treatments on the local structure, ionic conductivity, and battery performance of Li_5.5_PS_4.5_Cl_1.5_. It was found that a reduction in coherence length and particle size has opposite effects on the battery performance: a smaller particle size enhances battery performance due to a more uniform interface with the Li metal anode,^17^ higher interfacial area^16^ and reduced tortuosity and in turn higher partial transport.^16,18^ In contrast, the loss in coherence length has been reported to cause a reduction in ionic conductivity in Li_5.5_PS_4.5_Cl_1.5_, adversely affecting battery performance.^18^ Despite the decreasing ionic conductivity, the activation energy *E*_A_ remained almost constant suggesting no influence on the total energy landscape.

In addition to a fundamental interest in understanding dynamics in solid electrolytes, such investigations are also of practical relevance as the consideration of thermal load in solid-state batteries will become important upon commercialization. Solid-state batteries can present thermal hazards; for instance, charged lithium nickel manganese cobalt oxide (NCM) in contact with sulfide electrolytes can ignite spontaneously at temperatures as low as 100 °C, even under inert atmospheres.^20^ Additionally, while thermal runaway may be avoided, elevated temperatures accelerate decomposition reactions, shortening battery life, whereas low temperatures impair ionic conductivity, reducing capacity and rate performance.^20,21^ Studies investigating thermal conductivity of solid electrolytes are scarce, but unanimously report that these materials deviate significantly from the predictions of the phonon gas model traditionally used to describe temperature-dependent thermal conductivity in crystalline solids.^22–24^ In this model, lattice vibrations, quantized as bosonic quasiparticles called phonons, propagate through the material until they get scattered. In electronic insulators, scattering either occurs at defects, such as mass contrasts due to site disorder or partial occupancy, strain or grain boundaries, or *via* interactions with other phonons.^25^ Characteristic for the phonon gas model is a peak in thermal conductivity at low temperatures (typically below 50 K). At higher temperatures the thermal conductivity is expected to scale with *T*^−1^, representing classic Umklapp scattering.^25^ These temperature dependences can be found in most crystalline solids. However, the phonon gas model struggles to accurately describe thermal conductivity in materials whose mean free path length approaches the interatomic distances, *e.g.*, glasses,^26^ materials with pronounced anharmonicity,^22,27^ a sufficient degree of atomic disorder^28^ or large unit cells.^29^ In such materials, heat is conducted *via* a fundamentally different types of phonon, denoted as “diffusons”. Instead of propagating through the crystal, their conduction mechanism can rather be thought of as a random walk of thermal energy.^29^ Small interband spacing found in materials with a large unit cell and high phonon linewidths, evoked by anharmonic interactions, facilitate the overlap of phonon modes and promote diffuson-type thermal transport. As phonon linewidths increase with temperature, diffuson-type thermal transport increases with temperature too and saturates at high temperature.^22^ Both types of thermal transport are not exclusive but can contribute in parallel to a material's thermal conductivity. Different two-channel theories and models have been developed to separate the total thermal conductivity by the respective channels.^28–30^ The distinction between phonon gas and diffuson channel is heavily based on the characteristics of the underlying lattice vibrations. It is yet unclear how the previously observed change from Debye-like to liquid-like phonon dynamics observed in lithium argyrodites transfers to the thermal conductivity and which roles local disorder, strongly prevalent in lithium-argyrodites,^9^ and disrupted long-range structural correlation play.

To investigate these influences, utilizing various characterization techniques is required. *Via* X-ray diffraction experiments reduced particle size and the introduction of microstrain upon enhanced mechanical energy input in two exemplary argyrodites, Li_5.5_PS_4.5_Cl_1.5_ and Li_6_PS_5_Br, are revealed. The pair-distribution function (PDF) extracted from total scattering experiments additionally yields information about the short- and long-range local structures. Corresponding refinements quantify the decrease in coherence length and indicate increased local disorder. To probe interrelations of the local structure and loss of coherence, including diminished crystallite size, introduced strain, and structural disorder, with the diffusion coefficient, quasi-elastic neutron scattering (QENS) is performed, whose activation energies are compared with the electrochemical impedance spectroscopy (EIS) results. These experiments demonstrate that the reduction in coherence length strongly affects the ionic conductivity of Li_5.5_PS_4.5_Cl_1.5_ and Li_6_PS_5_Br, highlighting the importance of tailored processing procedures for electrolytes to maximize their ionic transport in fabricated solid-state batteries.

The post-synthesis treatment may not only influence the structure and ionic transport but also vibrations within the lattice. *Via* Raman spectroscopy and inelastic neutron scattering (INS), changes in the atomic frequencies and phonon density of states (DOS) are probed. Those experiments are validated by *ab initio* lattice dynamics calculations, which provide additional insights into the local frequency distribution. Based on these characterization studies, differences in the temperature dependence of the thermal transport are evaluated. Contrary to the ionic conductivity, thermal transport and vibrational properties are affected only to a minor degree by loss of coherence. To further deconvolute the effects of structural disorder and loss of coherence, the Li_6_PS_5_Br_*x*_I_1−*x*_ substitution series is additionally investigated, to discern the influence of disorder within the series. This structural disorder is found to heavily influence thermal conductivity, especially at low temperatures.

This work offers a localized view of lithium-ion vibrations in these materials, uncovering the anharmonic character that gives rise to the recently observed liquid-like dynamics. These dynamics, in turn, lead to intrinsically low lattice thermal conductivities, providing deeper insight into the fundamental nature of fast ionic conductors.

## Experimental section

### Lattice dynamics

The required ordering of the unit cell for density functional theory (DFT) calculations requires distributing 24 Li^+^ ions across 120 positions (T2, T5, and T5a), resulting in ≈10^25^ possible permutations, not considering the anion site disorder. To simplify this, the primitive unit cell, containing one formula unit, was used.^31^ As the number of ions on each site in the primitive cell must be an integer, the occupancies were adopted accordingly. Given that the primitive unit cell contains only one halide atom, only 0% or 100% anion disorder scenarios can be calculated. Here, 0% disorder was chosen, as it is closer to the experimentally found disorder. Additionally, Li_5.5_PS_4.5_Cl_1.5_ could not be calculated due to the non-integer numbers of Li, S, and Cl atoms. Li_6_PS_5_Cl was calculated instead. Considering the similarities of lattice dynamic properties of Li_6_PS_5_Br and Li_6_PS_5_Cl, properties of Li_5.5_PS_4.5_Cl_1.5_ can be approximated using Li_6_PS_5_Cl with high accuracy. Crystal structures of Li_6_PS_5_Br and Li_6_PS_5_Cl were taken from the literature.^9^ All structural orderings were cleaned of symmetrically equivalent duplicates and the remaining structures were relaxed by employing DFT. All DFT calculations were performed using the Vienna *Ab Initio* Simulation Package (VASP)^32–34^ and utilizing projected-augmented-wave (PAW) potentials.^35^ The exchange correlation energy was calculated using the PBEsol functional within the generalized gradient approximation (GGA).^36^ The PAW potentials used were Li_sv 10 Sep 2004 1s^2^2s^1^, P 06 Sep 2000 3s^2^3p^3^, S 06 Sep 2000 3s^2^3p^4^, Cl 06 Sep 2000 3s^2^3p^5^, and Br 06 Sep 2000 4s^2^4p^5^. During relaxation, the atomic positions and unit cell volume were allowed to relax. Details on computational parameters can be found in Table S1. The structure with the lowest energy was taken for further lattice dynamics calculations. The resulting lattice parameters and a comparison to experimentally found lattice parameters are given in Table S2. Finally, the phonopy package^37,38^ was utilized for generating the displacements and post-processing. Computational details on the force calculations are given in Table S3.

### Synthesis

Syntheses of Li_6_PS_5_Br_*x*_I_1−*x*_ (*x* = 0, 0.1, 0.2, 1) and Li_5.5_PS_4.5_Cl_1.5_ were performed under an argon atmosphere. The stoichiometric amounts of Li_2_S (Thermo Scientific, 99.9%), P_4_S_10_ (Sigma-Aldrich), LiCl (Alfa Aesar, 99%), LiBr (Alfa Aesar, ≥99%), and LiI (Thermo Scientific, 99.999%) were ground in an agate mortar for 15 min, pelletized, and sealed under vacuum in carbon-coated quartz ampoules. The ampoules were dried at 800 °C for 2 h under dynamic vacuum prior to use to remove any traces of humidity. All syntheses were conducted in tube furnaces set to a heating rate of 100 °C h^−1^ and naturally cooled after the reaction. Li_5.5_PS_4.5_Cl_1.5_ was synthesized by heating the precursors to 450 °C for 3 days twice. In between both runs, the sample was ground, pelletized, and sealed as described above. Li_6_PS_5_Br_*x*_I_1−*x*_ was made by heating the precursors to 550 °C for 2 weeks. After the syntheses, the products were hand-ground before further processing and characterization.

Samples of four different coherence lengths were synthesized by different post-synthesis treatment procedures.^18^ The first sample, referred to as pristine or simply XL (extra-long coherence length), received no further treatment. The second part with long coherence length (*L*) was shaken for 10 min at 40 Hz utilizing a Fritsch PULVERISETTE 23. 200 mg of pristine argyrodite and five 5 mm ZrO_2_ balls were placed in a 15 mL ZrO_2_ cup. The third and fourth processing methods included a Fritsch PULVERISETTE 7 premium line planetary ball mill. 1 g of pristine argyrodite was mixed with 10 g of milling media (5 mm ZrO_2_ balls) in an 80 mL ZrO_2_ cup. Each milling cycle consisted of 10 min of milling time at 500 rpm and 10 min of resting time. For one sample only a single cycle was performed, and the other one was milled for 24 cycles (4 h of total milling time). The resulting samples with medium and short coherence lengths are consequently referred to as M and S, respectively.

The samples for quasi- and inelastic neutron scattering experiments were made using ^7^Li enriched precursors (^7^Li_2_S, ^7^LiCl, and ^7^LiBr, Sigma Aldrich, ≥99 atom % ^7^Li, ≥99% purity). The masses were adopted to account for the altered molar masses. The synthesis and post-synthesis procedures remained the same. However, due to limited beam time and the cost of the precursors only the pristine and 4 h milled (XL and S) samples were investigated.

### Electrochemical impedance spectroscopy

Ionic conductivities were determined using potentiostatic electrochemical impedance spectroscopy. Pellets of 300 mg to 400 mg were pressed isostatically at 500 MPa for 40 min, resulting in relative densities of 83% to 88% (see Table S6). On either side of these pellets 100 nm thick gold electrodes were sputtered. The pellets were sealed in pouch cells and the electrodes contacted using Al current collectors. Impedance spectra were acquired in a temperature range from −120 °C to 60 °C and a frequency range from 25 mHz to 10 MHz employing an Alpha-A impedance analyzer (Novocontrol Technologies) with a root-mean-square excitation voltage of 20 mV. To ensure thermal equilibration each temperature was held for 1 h before starting the impedance measurements. The impedance spectra were fitted using the RelaxIS 3 software (rhd instruments). Where possible, the impedance response was fitted with a constant phase element (CPE) for the blocking behavior of the gold electrodes and an (R)(CPE) element for the bulk grain boundary contributions. For measurements with insufficient data points to fit the (R)(CPE) element, this element was replaced by an ohmic resistor.

### X-ray scattering

X-ray diffractograms were collected on a Stoe STADI P diffractometer in Debye–Scherrer geometry equipped with a Ge(111) monochromator and a Dectris MYTHEN2 1K detector. Samples were sealed in glass capillaries with a diameter of 0.5 mm. For the Williamson–Hall analyses and analyses of the amorphous content, Cu K_α1_ radiation (*λ* = 1.540562 Å) was used. The diffractograms were measured in a 2*θ* range from 10° to 90° with a step size of 0.015°. Williamson–Hall analysis was used to quantify the strain *ε* of the materials according to eqn (1).^39^1
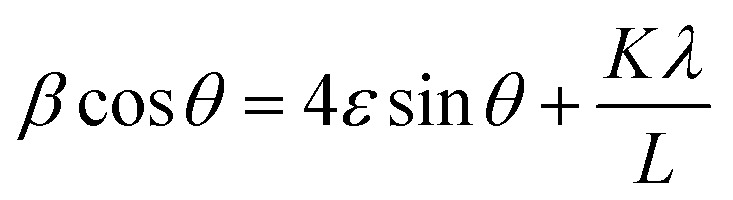


The peak broadening was quantified using the integral breadth *β* of each individual reflection of X-ray diffraction data. A pseudo-Voigt function was used to fit the intensity of each individual Bragg reflection as described elsewhere.^40^ The constant *K* was assumed to be 0.9, corresponding to spherical crystallites, while strain *ε* and crystallite size *L* were then extracted by linear regression in a Williamson–Hall plot. For the analyses of the amorphous content the argyrodite sample was mixed with silicon powder (20 wt%). Comparison of actual and Rietveld refined percentage by weight of argyrodite yields the argyrodite's amorphous fraction (Tables S3 and S4).

Low temperature X-ray diffractograms were measured in a 2*θ* range from 6° to 40° with a step size of 0.015° using Mo K_α1_ radiation (*λ* = 0.70932 Å). The sample temperature was controlled by employing a Cryostream 1000 (Oxford Cryosystems). For each sample short measurements were conducted in 4 K intervals, with prolonged measurements at specific temperatures.

For pair distribution function (PDF) analyses total scattering data were measured on a Stoe STADI P diffractometer in Debye–Scherrer geometry using Ag K_α1_ radiation (*λ* = 0.5594075 Å), a Ge(111) monochromator and four Dectris MYTHEN2 1K detectors.^41^ Samples were sealed in glass capillaries with a diameter of 0.5 mm and measured over a *Q*-range of 0.8–20.5 Å^−1^. Data reduction was done using PDFgetX3 (ref. 42) with a *Q*-range cutoff of *Q*_max_ = 15 Å^−1^. Small box modeling of the PDF was done using TOPAS academics V7.^43^ The data were fitted in an *r*-range of 1.8 Å up to 70 Å. For each sample the *r*-range was first limited to 10 Å and successively increased in 10 Å steps to 70 Å. The final model of an iteration was used as input for the subsequent *r*_max_ value. For each model the (1) scale factor, (2) correlated motion factor, (3) spherical diameter, (4) lattice parameters, (5) atomic positions, (6) isotropic atomic displacement parameters, and, in the case of Li_6_PS_5_Br, (7) the occupancies were refined.

### Quasi-elastic neutron scattering (QENS)

QENS measurements were performed using the BASIS backscattering spectrometer^44^ at the Spallation Neutron Source (SNS) at the Oak Ridge National Laboratory (ORNL). Due to limitations of beam time, only samples with the highest and lowest coherence length were investigated, *i.e.*, pristine and 4 h ball-milled samples. 5 g of ^7^Li enriched (>99% ^7^Li) argyrodite samples were filled under inert conditions into aluminum sample holders (1 mm-spaced double wall cylinder), sealed with aluminum foil as a gasket and loaded in a closed-cycle refrigerator with a hot stage. The pristine samples were measured at 60 K, 300 K, 350 K, 400 K, 500 K, and 600 K, whereas the ball-milled samples were measured at 250 K (only ^7^Li_6_PS_5_Br), 300 K, 325 K, and 350 K to avoid recrystallization of the samples. Additionally, brief measurements every 10 K were performed upon heating from 60 K to the maximum temperature. At 60 K, diffusion was assumed to occur on timescales slower than those accessible using BASIS. Consequently, data measured at this temperature were taken as sample-specific instrument resolution functions. Si(111) analyzers were used with a neutron wavelength centered at 6.4 Å and a chopper frequency of 60 Hz. This configuration allows access to a *Q*-range of 0.2–2 Å^−1^ with energy transfers of −120 μeV to 120 μeV. The data were reduced, normalized against a vanadium standard, and grouped into *Q* bins of 0.2 Å^−1^ width and energy bins of 0.8 μeV width using the Mantid software package.^45^ The *S*(*Q*, *E*) was fitted using a delta function *δ*(*E*) and a Lorentzian function with the half width at half maximum *Γ*(*Q*), representing the elastic signal and the quasi-elastic broadening, respectively. Both functions were centered around energy *E*_0_, convoluted with the resolution function *R*(*Q*, *E*), and scaled using the parameters *A* and *B*. Additionally, a linear background *C*(*Q*, *E*) was considered. Fitting of *S*(*Q*, *E*) was performed using the Dave software package.^46^2
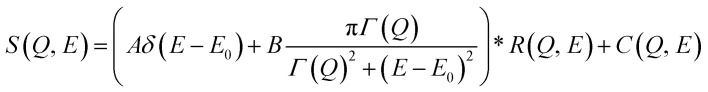


Data of the pristine samples were analyzed using the Chudley–Elliott model,^47^ capable of obtaining jump distances *d*, mean residence times *τ*, and thereby the diffusion coefficient.3
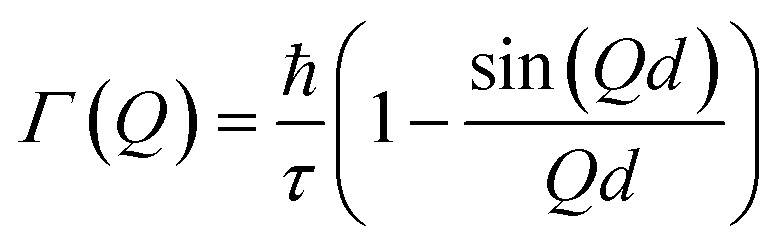


According to random walk theory, the diffusion coefficient is given by4
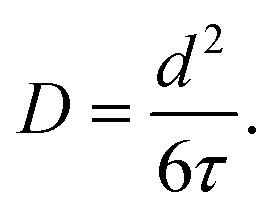


### Inelastic neutron scattering (INS)

Inelastic neutron scattering experiments were performed at the ARCS neutron time-of-flight spectrometer^48^ at the SNS, ORNL, using the exact same ^7^Li-enriched samples as those in the QENS experiments, *i.e.*, with the highest and lowest coherence lengths. Samples were loaded into ½ inch cylindrical aluminum cans. To eliminate peak broadening due to ionic diffusion all measurements were conducted at a sample temperature of 100 K. Scattering data were recorded for incident neutron energies of *E*_i_ = 15 meV, 40 meV, and 100 meV, a slit spacing of 1.5 mm, and chopper speeds of 240 Hz, 360 Hz, and 600 Hz, respectively. Li_5.5_PS_4.5_Cl_1.5_ was additionally measured at an incident neutron energy of 200 meV. At each energy the background of an empty aluminum can was collected and subtracted from the sample data after data reduction. Slight variations in detector efficiency were removed by normalization to a white beam V data set. For data reduction the Mantid DGS package was used.^45^ A measurement on a vanadium standard was used to correct the data for detector efficiency. The analysis of the phonon DOS was performed within the incoherent scattering approximation and corrected for the effect of multiphonon scattering in an iterative procedure using the multiphonon package.^49^ The neutron weighted simulated density of states (DOS) *g*_sim_(*E*) was calculated using the atom-projected calculated phonon DOS *g*_*j*_(*E*) of each element and by weighing it with respect to its total neutron scattering cross-section *σ*_tot_ and the atomic mass *m*_*j*_.5
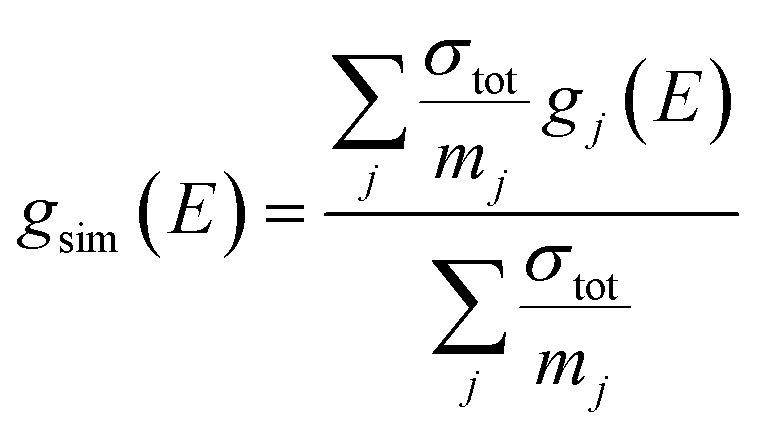


As no calculated phonon DOS for Li_5.5_PS_4.5_Cl_1.5_ was available, the phonon DOS of Li_6_PS_5_Cl was used and adopted for the different atomic composition in Li_5.5_PS_4.5_Cl_1.5_.

### Neutron powder diffraction

Samples of Li_6_PS_5_Cl, Li_6_PS_5_Br, and Li_5.5_PS_4.5_Cl_1.5_ were measured on the high-resolution powder diffractometer D2B at the Institut Laue-Langevin using neutrons with a wavelength of 1.594 Å and a 2*θ* range of 11° to 160°. 3 g of sample were loaded under an argon atmosphere into 8 mm diameter vanadium cans which were sealed with an indium wire. Samples below room temperature were cooled using a top-loaded cryostat. Neutron powder diffraction on Li_6_PS_5_I was performed at the POWGEN beamline^50^ at the SNS, ORNL. 2.5 g of Li_6_PS_5_I were loaded into a 6 mm vanadium can and sealed with a Cu gasket. Neutrons with wavelengths centered at around 0.8 Å were used for data collection. The sample environment was controlled using the POWGEN Automatic Changer.

### Rietveld refinements

Rietveld refinements against X-ray and neutron diffraction data were performed with the TOPAS Academic V7 software package.^43^ Refinements included (1) a scale factor, (2) background using a Chebyshev polynomial function, (3) a zero offset, (4) instrumental parameters, (5) peak shape parameters from the modified Thompson–Cox–Hastings pseudo-Voigt function,^51^ (6) lattice parameters, (7) site fractional coordinates, (8) occupancy factors, and (9) isotropic displacement parameters. Finally, all parameters were refined simultaneously to ensure stability of the parameters and a converged refinement. The quality of the refinements was assessed using the *R*_wp_ and the goodness-of-fit (GoF) values as indicators.

### Raman spectroscopy

Raman spectra were acquired using a SENTERRA II spectrometer by Bruker, equipped with a 532 nm laser. In-house made sample holders were used to protect the samples against ambient moisture. Each spectrum was measured with a laser power of 2.5 mW, an integration time of 5 s and 5 coadditions in a range of 50–1425 cm^−1^ with a resolution of 1.5 cm^−1^.

### Thermal conductivity

Thermal conductivities were measured with the Thermal Transport Option of a Physical Property Measurement System (PPMS) DynaCool (Quantum Design). Due to the low thermal conductivity samples were measured in two-probe lead configuration using disk shaped samples. Samples were first consolidated with a vice press using a 4 mm pressing tool and subsequently pressed isostatically at 500 MPa for 40 min. Flash sintered pellets were sealed in carbon-coated quartz ampoules (see the Synthesis section) inserted into a tube furnace preheated to the synthesis temperature, annealed for 15 min and then cooled naturally inside the furnace. Sample leads were attached using EPO-TEK® H20E two-component epoxy glue and a curing procedure of 80 °C for 3 h. Thermal conductivities were measured in a temperature range of 2 K to 300 K with a temperature rise of 3%.

High-temperature thermal conductivities of Li_6_PS_5_I were determined using an LFA 467 HyperFlash® setup (Netzsch). Below room temperature an MCT detector with a ZnS furnace window was used, whereas above room temperature an InSb detector with a sapphire furnace window was employed. Measurements were performed in an inert atmosphere of pure nitrogen with a flow of 100 sccm. The sample was spray-coated with a graphite layer to enhance absorption and emission of infrared light. Detection time and signal amplification were optimized automatically during the measurement. Three measurements were performed at each temperature (five measurements at 173 K) and the detector signal is fitted with an improved Cape–Lehman model.^52,53^ The obtained thermal diffusivity *α* can be converted to thermal conductivity *κ* using isobaric heat capacity *c*_p_ and density *ρ*.

6 *κ* = *α* × *c*_p_ × *ρ*

The isobaric heat capacity was approximated using the isochoric heat capacity obtained from density functional theory-based lattice dynamics calculation. The density was determined geometrically.

## Results & discussion

Li_6_PS_5_Br and Li_5.5_PS_4.5_Cl_1.5_ represent two of the most widely studied argyrodite-type solid electrolytes in the field of lithium solid-state batteries. While their fundamental properties have been extensively characterized in the as-synthesized, pristine state, the influence of post-synthetic processing, *e.g.*, for cathode composite fabrication, remains insufficiently understood. In this study, four samples of each argyrodite are subjected to varying degrees of mechanical energy input after the synthesis, to systematically investigate the influence of structural coherence on the ionic and thermal transport. Where required, comparative data for both compounds are provided. In cases where only one dataset is displayed in the main text, the results for Li_6_PS_5_Br are shown, with the corresponding data for Li_5.5_PS_4.5_Cl_1.5_ available in the SI.

### Loss in coherence

**Fig. 2:**
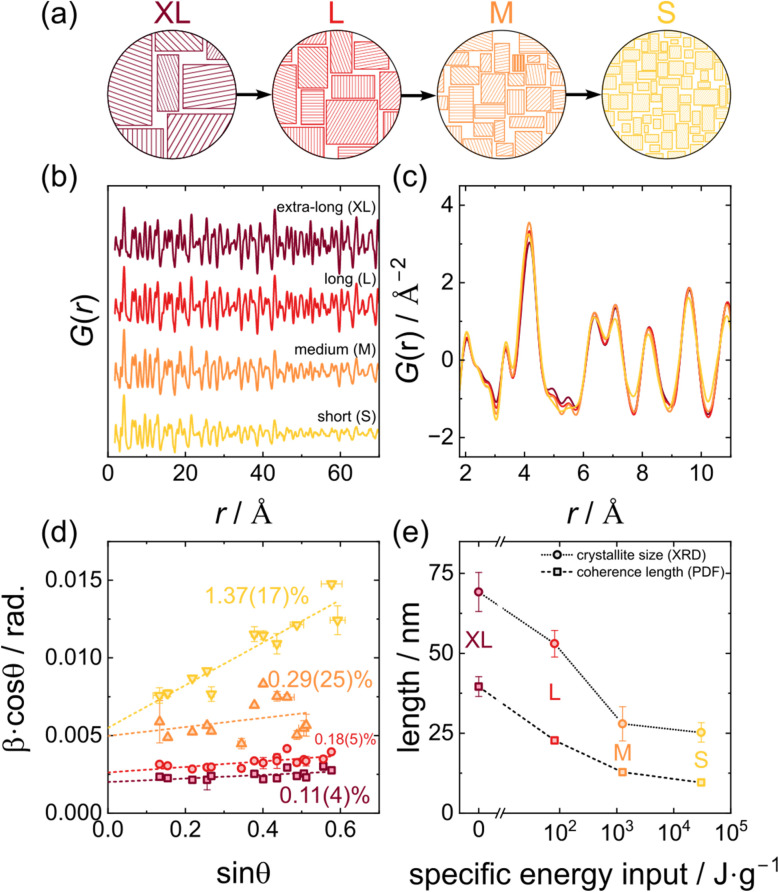
(a) Schematic reduction of crystallite size and coherence length across the differently labeled samples. (b) Long- and (c) short-range *G*(*r*) of Li_6_PS_5_Br for the different post-synthesis processing methods indicating the amount of local disorder and the loss in coherence. The peaks at the lowest distances of 2.1 Å and 3.4 Å can be assigned to P–S and S–S bonds of the PS_4_^3−^ unit, respectively. (d) Williamson–Hall analysis of Li_6_PS_5_Br samples with increasing amounts of strain and lower crystallite size, indicated by the higher *y*-axis intersect for samples with lower coherence lengths. The XRD patterns depicting the peak broadening are given in Fig. S7. (e) Coherence length as obtained from fits of *G*(*r*) and crystallite size from the Williamson–Hall analysis as a function of energy input. Although both methods differ by up to a factor of 2, the general trend and order of magnitude agree for both methods. An analogous plot for Li_5.5_PS_4.5_Cl_1.5_ is given in Fig. S1.

To correlate changes in the materials dynamics with the coherence length, first structural details in Li_6_PS_5_Br and Li_5.5_PS_4.5_Cl_1.5_ are analyzed by total scattering and Williamson–Hall analyses. The influence of post-synthesis processing on both the average and local structure and on the coherence length can be analyzed *via* the reduced pair distribution function *G*(*r*). All *G*(*r*) exhibit a decay of intensity (structural coherence) at increasing interatomic distances *r*. As previously reported by Maus *et al.*,^18^ this decay is intensified with enhanced energy input of the post-synthesis processing (Fig. 2b and S1a, refinements shown in Fig. S3 and S5). In the following, the labels “XL”, “L”, “M” and “S” are used, corresponding to the respective coherence length (extra-long, long, medium and short) of each processing method (Fig. 2a). The reduction in coherence is corroborated by Williamson–Hall analyses (Fig. 2d and S1c), which enable the deconvolution of crystallite size and strain contributions induced during processing, rather than providing only a total coherence length. While negligible strain is observed in the XL samples, increasing the energy input by employing different post-synthesis processing methods and thereby reducing coherence length introduces more and more strain. At the same time, enhanced energy input also leads to a reduced crystallite size, confirming the trend of diminished coherence lengths (Fig. 2e and S1d). Specific energy inputs were taken from previous simulations.^18^ Owing to the minimal peak broadening in the Bragg reflections used for the Williamson–Hall analyses and the gradual coherence decay observed in the *G*(*r*), both techniques face limitations in reliably quantifying large crystallite sizes and coherence lengths for the more crystalline compounds, respectively, resulting in noticeable uncertainties for high values.

Less significant differences of the *G*(*r*) between the different processing methods are observed at low distances (Fig. 2b and S1b) demonstrating that the processing does not affect the local structure of the materials. In both argyrodites, almost no differences can be seen at the shortest bonds present in the materials: the P–S bond within the PS_4_^3−^ tetrahedra and the Li^+^–X^−^/S^2−^ distances. Due to the small scattering cross section of lithium, the latter is only visible as a small peak in Li_5.5_PS_4.5_Cl_1.5_ and shoulder in Li_6_PS_5_Br. Thus, even for short coherence length the first coordination sphere in the argyrodite is preserved. Despite the well-known structure of lithium argyrodites, refining the short-range *G*(*r*) (Fig. S2 and S4) poses significant difficulties, *e.g.*, the observed peak at around 3.4 Å cannot be fitted using the standard cubic model. It has been suggested that a tilting of the PS_4_^3−^, resulting in a symmetry reduction, could fit this distance.^54,55^ At larger distances, however, the lowest-coherence sample in Li_6_PS_5_Br and the two samples with lowest coherence in Li_5.5_PS_4.5_Cl_1.5_ exhibit broader peaks with reduced intensity compared to the pristine counterpart (Fig. S6). Consequently, the fitting of these data required higher atomic displacement parameters, which is indicative of increased disorder in the local structure. Despite this disorder, diffraction experiments with silicon as an internal standard revealed that in all samples almost no amorphous content is present (Tables S4 and S5). So, while the post-synthesis procedures lead to a loss of coherence, reduced crystallite size, strain, and local disorder, they do not amorphize the solid electrolyte.

### Li^+^ ion dynamics and diffusion

The ionic conductivity of solid electrolytes is a critical parameter governing the performance of solid-state batteries and is influenced by multiple structural and microstructural factors, including coherence length and the local structure.^18^ To assess the impact of the different post-synthesis processing procedures on the ionic conductivity of Li_6_PS_5_Br and Li_5.5_PS_4.5_Cl_1.5_ electrochemical impedance spectroscopy (EIS) was performed. Spectra of all samples throughout the entire temperature range were well resolved and could be fitted with either an (R-CPE)-CPE or R-CPE equivalent circuit. Fits of data measured at 173 K and 298 K are given in Fig. S9–S12. *α*-Values of the CPEs of close to unity are indicative of almost ideally capacitive behavior, *i.e.*, perfect blocking behavior of the electrodes and uniform relaxation times. Across all samples, the Nyquist plots displayed a single, well-defined semicircle, preventing the deconvolution of bulk and grain boundary contributions. Therefore, all values reported correspond to total ionic conductivities.

**Fig. 3:**
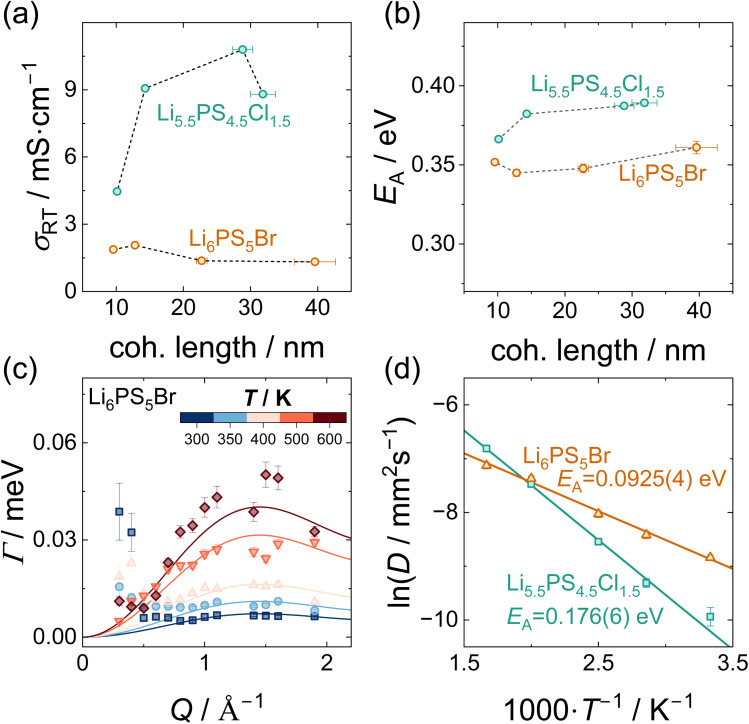
(a) Room-temperature ionic conductivities measured with impedance spectroscopy with respect to the coherence length. (b) Activation energies derived from temperature-dependent impedance spectroscopy. (c) *Q*-Dependence of the Lorentzian linewidth at various temperatures for Li_6_PS_5_Br and corresponding fits using the Chudley–Elliott model. *Q* bins with considerable contributions by Bragg peaks were excluded and are not shown in this plot. The corresponding plots for Li_5.5_PS_4.5_Cl_1.5_ can be found in Fig. S15. (d) Arrhenius plot of the diffusion coefficients obtained from the fits with the Chudley–Elliott model. Both argyrodites exhibit an Arrhenius-type behavior with higher activation energy found for Li_5.5_PS_4.5_Cl_1.5_.

**Fig. 4:**
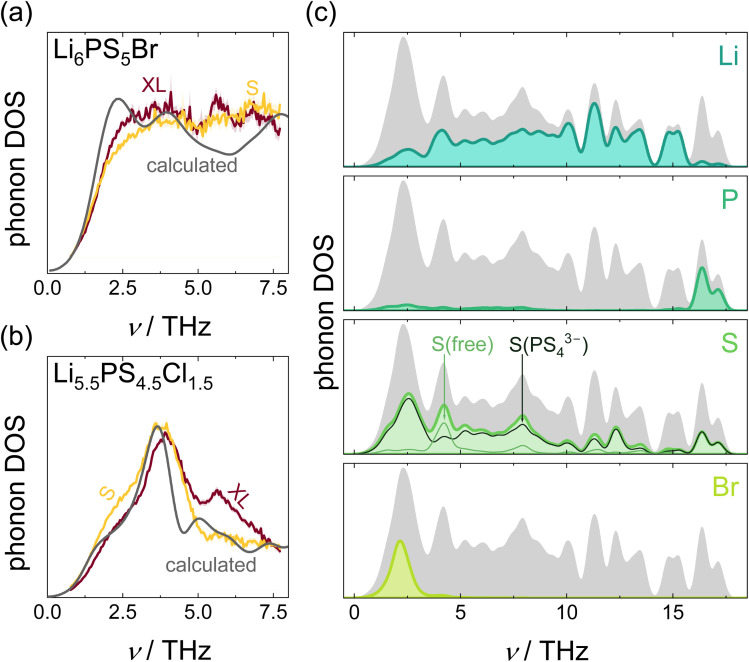
(a) and (b) Phonon DOS at 100 K as obtained from inelastic neutron scattering experiments using neutrons with 40 meV incident neutron energy and the modelled neutron-weighted phonon DOS. “XL” and “S” denote the coherence length of the respective sample. (c) Calculated atom-projected phonon density of states of Li_6_PS_5_Br. The gray area represents the total phonon DOS. The corresponding plot for Li_6_PS_5_Cl can be found in the SI (Fig. S19a).

In both argyrodites, the room-temperature ionic conductivity changes non-monotonically with respect to the coherence length. For both materials, moderate reductions in coherence length, associated with milder processing, lead to a slight increase in ionic conductivity (Fig. 3a). However, with further reduction in coherence length, resulting from harsher treatments, the conductivity decreases. The observed maximum in ionic conductivity might be caused by two opposing effects. First, previous studies have shown that smaller particle sizes result in better transport properties in cathode composites.^16,56^ Although particle size and crystallite size are not the same and in principle independent of each other, the processing procedures not only decrease crystallite size but also particle size,^18^ which consequently results in an increased ionic conductivity. Second, the increase in local disorder at small *r* observed for low coherence lengths, especially in Li_5.5_PS_4.5_Cl_1.5_, is believed to impede ionic transport as it alters inter-cage distances important for long-range ionic transport and thereby disrupts the interconnected 3D network of lithium-ion conduction pathways.^9,10^ In combination, these two opposite effects can result in the observed increase in ionic conductivity due a to reduction in particle size for mild processing, while for higher energy input, the reduction in crystallite size dominates, reducing ionic conductivity. Notably, in the chloride-rich argyrodite, the conductivity maximum is reached at comparatively higher coherence lengths, and the subsequent decline in conductivity is more pronounced than that in the bromine analogue. This drop in ionic conductivity for low coherence lengths is consistent with previous reports for Li_5.5_PS_4.5_Cl_1.5_.^18^ The less severe reduction of ionic conductivity for the bromide argyrodite could be linked to the strain introduced, which has been shown to enhance ionic conductivity in this system.^40^ Throughout the series of processing conditions, the activation energy that remains is only minimally affected with changes of less than 0.03 eV (Li_5.5_PS_4.5_Cl_1.5_ up to ≈−6%, Li_6_PS_5_Br up to ≈−4.5%) (Fig. 3b). As no deconvolution of bulk and grain boundary contributions is possible (see Fig. S9 and S12), it remains elusive if the changes in ionic conductivity are caused by a changing prefactor of bulk transport, by a change in grain-boundary resistances or by both. However, based on the unchanged first coordination sphere (Fig. 2c), virtually identical frequencies as observed with Raman spectroscopy (Fig. S13), and similar phonon densities of states (Fig. 4a and b), jump distance, attempt frequency, and migration entropy are not expected to change strongly, keeping the prefactor of bulk transport relatively constant. Thus, the grain-boundary resistance is assumed to be the main contributor to a change in ionic conductivity.

To gain further insights into the (change of) diffusion mechanism and ion dynamics in the argyrodites, quasi-elastic neutron scattering (QENS) experiments were conducted for comparison of the XL and S samples, corresponding to samples with the highest and lowest coherence length, respectively. X-ray diffraction analyses carried out on the low-coherence sample after sequential heating revealed the onset of recrystallization at approximately 373 K.^18^ Consequently, the QENS measurements on those samples were restricted to temperatures below this threshold, with a maximum of 350 K. By interacting with diffusing lithium ions, neutrons can gain or lose energy, resulting in a broadening of the elastic signal. This broadening, indicative of ionic motion, becomes apparent above 250–300 K, manifesting as an onset in quasi-elastic intensity (Fig. S14a and b).^57^ With increasing temperature, this broadening becomes significantly more pronounced as diffusion becomes faster and more ions become mobile. By integrating the quasi-elastic and background scattering intensity at different temperatures and normalizing to the lowest temperature (here 60 K), the onset of diffusion can be identified. As higher quasi-elastic intensity corresponds to an enhanced fraction of mobile ions, a qualitative comparison of the temperature-dependent fraction of mobile ions within the material can be performed (Fig. S14a and b). The onset of diffusion in XL Li_6_PS_5_Br occurs approximately 30 K earlier than that in XL Li_5.5_PS_4.5_Cl_1.5_. Although the room-temperature conductivity of Li_5.5_PS_4.5_Cl_1.5_ is higher than that of Li_6_PS_5_Br, the lower activation energy of the bromine argyrodite allows for higher conductivity at low temperatures, which is confirmed by the earlier onset of quasi-elastic intensity. For both materials, lower relative intensities above 300 K are observed for the samples with low coherence length compared to highly coherent samples. However, given the short measurement time at each temperature step, these differences are possibly within the experimental uncertainties.

The unprocessed, pristine samples can be heated to significantly higher temperatures than the low coherence samples, giving rise to a more intense (Fig. S14a and b) and broader (Fig. S14c) quasi-elastic signal, which can be fitted using a Lorentzian function. An exemplary fit of the entire signal according to eqn (2) is given in Fig. S14d. The *Q*-dependence of the linewidth of this Lorentzian allows a more detailed analysis using the Chudley–Elliott model.^47^ Although in general this model allows the jump distance of the ions to be obtained, the extraction of the jump distance is less reliable and more error-prone than the fit of the relaxation time. So, previously reported crystallographic inter-cage jump distances of 2.8 Å^11^ and 3.1 Å^9^ for Li_5.5_PS_4.5_Cl_1.5_ and Li_6_PS_5_Br, respectively, were used to guide the fit (Fig. 3c and S15). A previous study investigating the ion dynamics of closely related Li_6_PS_5_Cl confirmed that the QENS signal is dominated by the inter-cage dynamics.^14^ Fits in which the jump distance was refined too yield jump distances comparable to crystallographic jump distances (Fig. S16 and Tables S7, S8). Using the fitted mean residence times and the crystallographic jump distances, the Li^+^ diffusion coefficients were calculated by employing eqn (4). The obtained diffusion coefficients follow closely an Arrhenius-type behavior with activation energies of 0.176(6) eV for Li_5.5_PS_4.5_Cl_1.5_ and 0.0925(4) eV for Li_6_PS_5_Br (Fig. 3d). At the lowest temperatures, the linewidth is almost independent of *Q*, which could indicate localized diffusion between two lattice sites. This can be expected as the intra-cage doublet jump between two T5 sites is associated with a lower activation energy than the inter-cage T2–T2 jumps and thus particularly favored at low energies.

Since QENS probes dynamics on shorter length scales than impedance spectroscopy, the activation energies derived from QENS are significantly lower than those from impedance measurements (typically 0.3 eV to 0.4 eV) (Fig. 3b). Additionally, QENS selectively captures local ion hopping processes and is thus insensitive to microstructural features such as grain boundaries, which can alter the apparent activation behavior. Despite these methodological differences, the relative trend is consistent: a higher activation energy is found for Li_5.5_PS_4.5_Cl_1.5_ compared to Li_6_PS_5_Br. Given the multitude of nuclear magnetic resonance (NMR) methods and their vastly different timescales and methodologies, comparing the activation energy and diffusion coefficients requires careful consideration.^58^ Nevertheless, activation energies obtained from QENS match those obtained by NMR methods probing short timescales and fast dynamics (see Tables S9 and S10 for a detailed comparison) and are consistent with the previously reported 0.11 eV for Li_6_PS_5_Cl extracted from QENS data.^14^

### Lattice dynamics on global and local scales

The fast ion dynamics of lithium ions culminates in a breakdown of phonon modes at high temperatures, preventing classic solid-state lattice dynamics from accurately capturing phonons in these systems.^14^ At low temperatures, however, lattice dynamics on a local scale can provide insights into ion dynamics and transport beyond structural arguments and results inferred from measurements of diffusion or conductivity *via* EIS, QENS or NMR. Previous work has employed different approaches to track changing lattice dynamics within the substitution series allowing ionic transport to be better understood,^59^ by demonstrating that vibrations in the direction of ionic transport possess extraordinarily low frequencies,^22^ and elucidating the role of different degrees of freedom in ionic transport.^23,60^ Therefore, here, the lattice dynamics of Li_6_PS_5_Br and Li_5.5_PS_4.5_Cl_1.5_ are characterized by both experimental and computational methods. Experimentally derived phonon densities of states (phonon DOS) from inelastic neutron scattering reflect the total DOS and cannot be decomposed into atom-, site-, or direction-specific contributions, but may capture the microstructural effects. *Vice versa*, density functional theory (DFT) calculations performed under periodic boundary conditions model idealized single crystals and thus exclude microstructural influences. While this limits direct comparability to real samples in terms of coherence length and disorder, calculated phonon DOS enables decomposition of the vibrational spectra by atomic species, crystallographic sites, and even the spatial direction, offering detailed, local insights into vibrational modes relevant to ionic transport. For a holistic study of vibrations in argyrodites on a global and local scale, inelastic neutron scattering experiments with lattice dynamics calculations are combined here.

Despite the differences observed in X-ray diffraction measurements and the reduced coherence length, the phonon DOS of XL and S samples are similar, both for the bromine (Fig. 4a, S17a and S18a) and the chloride (Fig. 4b, S17b and S18b) argyrodite, and independent of the incident neutron energy (15 meV for Fig. S17, 40 meV for Fig. 4a, and 100 meV for Fig. S18). This outcome aligns with prior studies^61,62^ showing similar phonon DOS in fully amorphous and fully crystalline forms of the same material. Given the high scattering cross-section of Cl^−^ ions and the confined frequency range of the corresponding vibrations, the neutron-weighted DOS (NWDOS) of Li_5.5_PS_4.5_Cl_1.5_ exhibits a peak at low frequencies, which was also observed in the experimental phonon DOS. Nevertheless, the experimentally measured DOS of both argyrodites contains a peak at 14 THz (Fig. S18), which is not predicted by the calculated NWDOS, but was previously also found in Li_6_PS_5_Cl and linked to vibrations of PS_4_^3−^ tetrahedra.^14^

Raman spectroscopy offers, compared to the INS-measured phonon DOS, a much higher frequency resolution, enabling small frequency changes to be detected. This high resolution enabled detection of a slight red-shift of approximately 0.04 THz for the ball-milled samples (Fig. S13a), corresponding to an almost unnoticeable softening of the lattice. Although this shift is close to the resolution of the instrument (0.045 THz), fits of the peak shape confirmed this shift. As the high frequency vibrations of the PS_4_^3−^ units (at 17 THz to 18 THz, corresponding to approx. 570 cm^−1^ to 600 cm^−1^) are Raman-active, they can be used to check the accuracy of the atom-projected DOS of phosphorus, revealing good agreement between measured and calculated frequencies (Fig. S13b).

Despite the shortcoming of not capturing microstructural effects, given the similarity of phonon DOS between crystalline and amorphous samples reported before^61,62^ and found here between samples of different coherence lengths, the lattice dynamics calculations can be used as a proxy for both the high- and low-coherent samples. Atom projections of the phonon DOS reveal vibrations of lithium and sulfur ions throughout the entire frequency range, whereas the halide and phosphorus ions contribute to the DOS in a much narrower frequency range. These contributions are located at vastly different frequencies though. Vibrations of P^5+^ ions are located almost exclusively at the upper end of the frequency spectrum and overlap only with the DOS of PS_4_^3−^-bonded sulfur ions, suggesting almost exclusive vibrations of PS_4_^3−^ tetrahedra (Fig. 4c and S19a).^59^ In contrast, the halide ions, Cl^−^ and Br^−^, are coordinated by comparably large cages of Li^+^ ions, in contrast to the much smaller PS_4_^3−^ tetrahedra. Since the vibrational frequency scales with the square root of the force constant divided by the atomic mass, this much softer bonding environment, associated with low force constants, and the higher mass in the case of bromide, cause the halide vibrations to be found in the low frequency region.

**Fig. 5:**
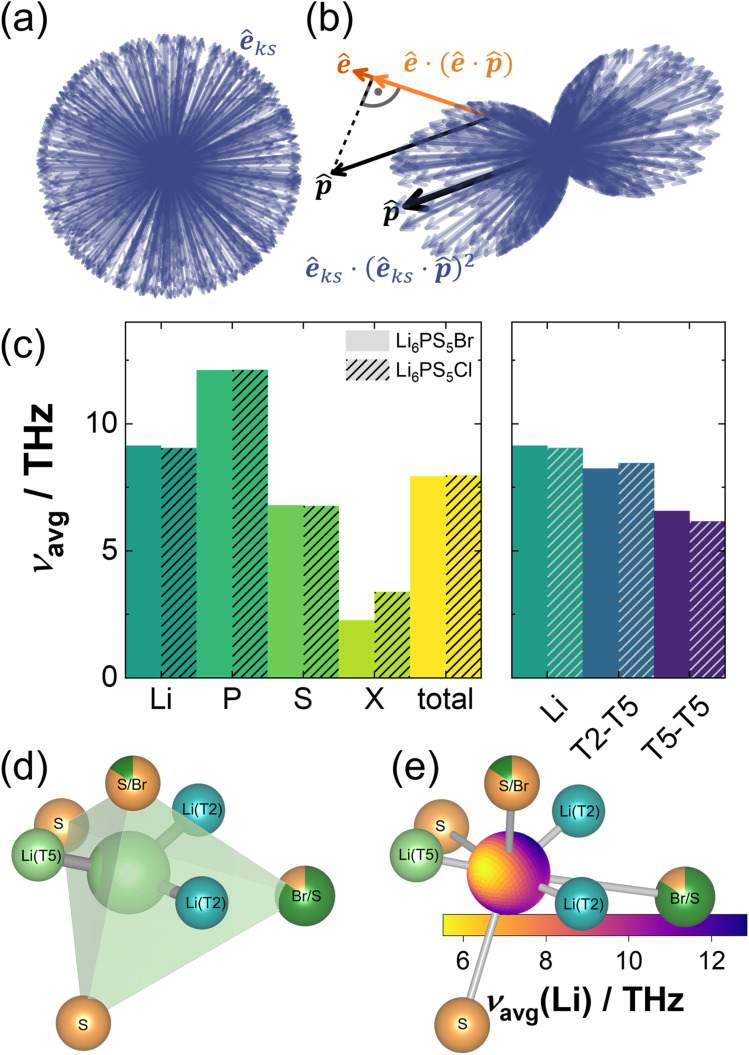
(a) Exemplary distribution of eigenvectors **ê** when not projected in a specific direction. Each eigenvector is a function of the *q*-point *k* and the branch index *s*. (b) Effect of projecting the phonon DOS along the direction **p̂**, both over all eigenvectors and, in more detail, for a single selected eigenvector. For visual clarity all eigenvectors are normalized to unit vectors. (c) Average atom-projected vibrational frequencies of Li_6_PS_5_Br and Li_6_PS_5_Cl. X denotes the respective halide ion. (d) Coordination environment of the T5 site in Li_6_PS_5_Br. (e) Distribution of average frequencies in all spatial directions. The orientation is the same as that in panel (d). A respective plot for Li_6_PS_5_Cl can be found in the SI (Fig. S19).

These differences in site-projected phonon DOS translate to vastly different average vibrational frequencies, occasionally also referred to as phonon band centers.^63^ Average vibrational frequencies of a compound and the species therein serve as an indicator of the “softness” of the material and its respective sublattices. Lower average vibrational frequencies are often associated with enhanced ionic conductivity, as they correlate with reduced energy barriers for ion migration. Accordingly, lattice softening is a commonly pursued strategy to improve ion transport properties.^59,64^ Here, the average frequencies in both materials and their respective elements are found to be almost identical (Fig. 5c). The difference in vibrational frequency between both halide ions can be explained almost entirely by their mass difference. Given these similarities between argyrodites with different halides, it can be assumed that the phonon DOS of Li_6_PS_5_Cl is a good approximation for the phonon DOS of Li_5.5_PS_4.5_Cl_1.5_. High charge and a tight coordination environment within the PS_4_^3−^ tetrahedra cause phosphorus to exhibit the highest average frequencies of 12.1 THz. Despite being bonded to the relatively stiff PS_4_^3−^ tetrahedra, the average frequencies of the sulfide ions forming PS_4_^3−^ tetrahedra are only slightly above those of the “free” sulfide ion (6.1 THz and 7.0 THz, respectively). Because the frequency scales with the force constant, low frequencies correspond to high thermal displacements and indicate loose bonding. Taking the low mass of lithium into account, the average lithium-ion frequencies, which are only slightly above average compared to the total material, suggest below-average bond strengths.^59^ Based on neutron powder diffraction data in this study and in the literature,^9^ Li^+^ ions were found to occupy multiple sites (T2 and T5 in Li_6_PS_5_Cl and T2, T5, and T5a in Li_6_PS_5_Br). However, during relaxation all lithium ions were found to relax onto T5 positions. As these DFT calculations are performed at 0 K, this relaxation behavior aligns with experimental observations made here and in the literature,^9^ which found higher occupations of the T5 site at low temperatures, *i.e.* the T5 site is energetically slightly favored over the T2 and T5a sites. Each T5 position is coordinated by one halide, one “free” sulfide, and two PS_4_^3−^-bonded sulfide sites (Fig. 5d and S19b). This tetrahedron is face-sharing with another T5 tetrahedron and two T2 tetrahedra (Fig. 1b). The calculated phonon DOS can be projected not only on individual sites but also in specific directions. To do so, contributions of phonon modes are weighted by the length of the projection of the site- and mode-specific eigenvector on the projection vector.^38^ Eigenvectors orthogonal to the direction of projection are therefore neglected, whereas eigenvectors parallel to the projection vector possess high weighting factors (compare Fig. 5a and b). Projecting the phonon DOS in the direction of the neighboring lithium ion sites, *i.e.*, in the direction of ionic transport, reveals a significantly reduced average vibrational frequency, predominantly for the T5–T5 jump (Fig. 5c). Low force constants towards the adjacent T5 site correspond to a shallow potential and high thermal displacement parameters, consistent with experimental reports which find Li^+^ occupancy at the bottleneck of the T5–T5 position for the bromide argyrodite indicative of a low energy barrier for this jump. This low activation barrier was also confirmed *via* nudged elastic band calculations on Li_6_PS_5_Cl.^65^

The radial distribution of average vibrational frequencies not only holds information on the average frequency in the jump direction, but also in every other spatial direction (Fig. 5e and S19c). Due to the loss of symmetry while ordering the primitive unit cell, each of the six Li^+^ ions within the primitive unit cell faces a slightly different local environment, leading to a slightly different radial frequency distribution. Fig. 5e and S19c show therefore only chosen representatives for the six individual positions. Irrespective of the halide ion in the structure, they all have in common that the central Li^+^ ion is located only slightly out of the plane formed by the adjacent Li^+^ sites. Regions of low average vibrational frequency are found predominantly along that plane, especially towards the T5 position, whereas regions of high frequency are rather found perpendicular to that plane. These low frequencies in the jump direction result in high thermal displacement parameters, typically indicative of fast ionic conductors. The deviations between harmonic and anharmonic potentials increase with enhanced displacements from the equilibrium position. Hence, the low frequencies in the jump direction found herein suggest a strongly anharmonic, shallow potential energy landscape, corroborating findings by Ding *et al.*,^14^ who showed that such a potential energy landscape leads to liquid-like ion dynamics and ultimately a non-zero DOS at zero-frequency at elevated temperatures (see the schematic in Fig. 1c) in lithium argyrodites.

### Effect of coherence length on thermal conductivity

**Fig. 6:**
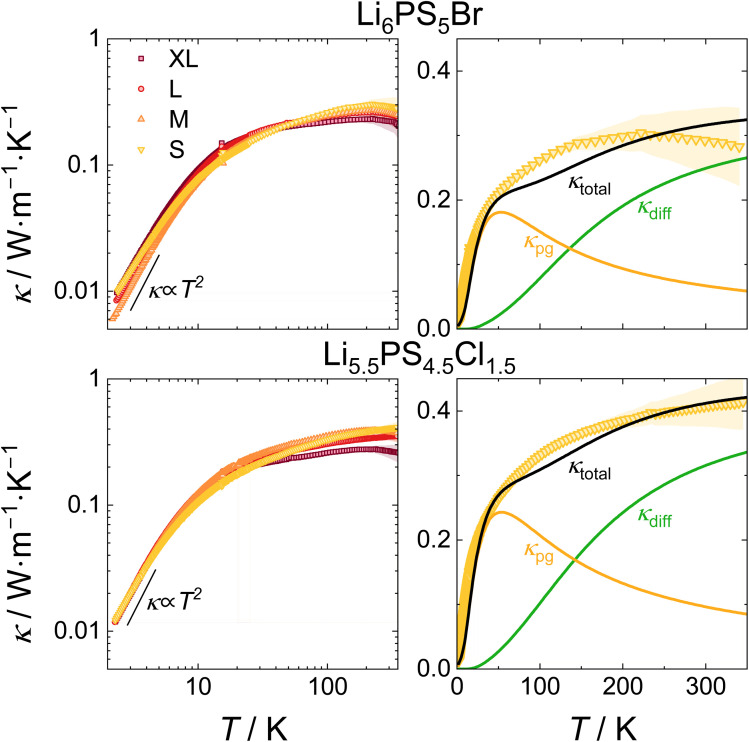
Thermal conductivities of Li_6_PS_5_Br and Li_5.5_PS_4.5_Cl_1.5_ processed with different coherence lengths. At the lowest temperatures, below 5 K, thermal conductivities scale approximately with *T*^2^ (left panels). The samples with the lowest coherence length were fitted using an analytical two-channel model. At low temperatures, significant phonon gas contributions were found (right panels).

Despite the overall similarity in phonon DOS found for pristine samples and those with reduced coherence length and increased disorder, the thermal conductivity can still vary between these materials. Especially at low temperatures reduced crystallite sizes and increased disorder can function as defects at which phonon gas like phonons can get scattered.^25^ Therefore, investigating thermal transport at cryogenic temperatures is paramount to assess potential changes in the thermal transport of both argyrodites with respect to their coherence length. At high temperatures, the shift from Debye-type to liquid-like lattice dynamics^14^ holds the potential to dominate the magnitude and temperature dependence of thermal transport. Different ion mobilities may influence this transition and thus the thermal conductivity. Samples of Li_6_PS_5_Br and Li_5.5_PS_4.5_Cl_1.5_ were measured over a temperature range of 2 K to 350 K (Fig. 6). The maximum temperature was set at 350 K, as the broadening of the reflections (Fig. S7) and with this the effects of the post-synthesis processing were found to anneal out at temperatures as low as 373 K. Across the entire temperature range studied, low thermal conductivities were observed. Above 100 K, the thermal conductivity remains nearly constant, reflecting strong diffuson contributions at higher temperatures. This behavior is consistent with previous findings that reported a flat thermal conductivity profile for argyrodites extending beyond 350 K.^66^ At low temperatures, no distinct peak in thermal conductivity is observed; instead, the thermal conductivity increases sharply below 50 K before flattening out. Below 5 K, thermal conductivities scale approximately with *T*^2^, typically for dominant grain-boundary scattering in polycrystalline materials.^67^ A subtle trend may be discernible in which the reduced coherence lengths lead to a diminished increase in thermal conductivity with rising temperature. To approximate the contribution of each type of thermal transport, an analytical two-channel model is applied to the samples with the lowest coherence length (right panels in Fig. 6; for details see Section S7).^28^ This model successfully reproduces the experimentally measured thermal conductivities and demonstrates that neither purely diffuson-like nor phonon gas-like transport alone can fully account for the observed thermal transport behavior at low temperatures. Instead, thermal transport in the cryogenic regime seems to be primarily governed by phonon gas-like transport. At temperatures above ≈140 K diffuson-like conduction begins to dominate. The pronounced anharmonicity of lattice vibrations in argyrodites demonstrated here and by Ding *et al.*,^14^ as well as the liquid-like dynamics of lithium ions at higher temperatures, accompanied by a breakdown of the Debye-type phonon DOS, disrupts phonon propagation and is believed to enhance phonon linewidth. Although the transition to liquid-like dynamics occurs at around 400 K, its influence extends into the temperature range over which thermal conductivity was measured (up to 350 K).^14^ These factors contribute to the dominance of diffuson-type and the low magnitude of thermal transport at elevated temperatures.^22,68^ Additionally, these findings may also explain the observed reduction in the sharpness of the conductivity increase in samples with reduced coherence lengths. Enhanced disorder, smaller crystallites, and the introduction of strain can function as additional defect sites that scatter phonons, increasing resistance to heat transport and reducing phonon gas-type thermal conductivity. In contrast, structural effects such as anion disorder and strain might be expected to enhance diffuson-type transport: variations in the local coordination environment of atoms change the (local) frequency of vibrations, which broadens the phonon linewidth and thereby increases diffuson-type thermal transport. However, the minor changes observed in the pair distribution function, primarily affecting the second and higher coordination spheres, indicate that the associated increase in vibrational disorder may be insufficient to enhance diffuson transport within the sensitivity limits of the measurements. Overall, no strong influence of the coherence length on the thermal transport is found, further corroborating the dominance of local vibrations and anharmonicity on the transport.

### Effect of structural disorder on thermal conductivity

While the effect of coherence length on thermal conductivity is found to be rather minor, previous reports on thermal conductivity in substitution series of solid electrolytes^22,28^ have demonstrated much more significant changes upon structural ordering. Typically, phonon peaks are observed for fully ordered endmembers and a suppression of such within the substitution series is found.^28^ In the family of lithium argyrodites, the iodine argyrodite Li_6_PS_5_I exhibits even at room temperature a fully anion ordered host lattice due to the significant size mismatch between sulfur and iodine ions, which suppresses site disorder.^9^ Upon cooling, Li_6_PS_5_I undergoes a phase transition to a fully ordered monoclinic phase (*Cc*, space group no. 9).^69^ Within the Li_6_PS_5_Br_*x*_I_1−*x*_ substitution series, partial substitution of iodine by bromine progressively introduces structural Br^−^/I^−^ and potentially Br^−^/S^2−^ disorder into the host lattice, making it optimally suited to investigate the effect of structural disorder on the thermal conductivity in lithium argyrodites. Neutron and X-ray powder diffraction are used to determine the presence of a phase transition and, in the case of a phase transition, the structure of the low temperature polymorph and the phase transition temperature. Li_6_PS_5_I, Li_6_PS_5_Br_0.1_I_0.9_, and Li_6_PS_5_Br_0.2_I_0.8_ all exhibit a phase transition to a monoclinic phase. The phase transitions stretch over a certain temperature range, which gets wider and shifts to lower onset temperatures as the bromine content increases (Fig. S20–S24). Similar phase transitions in Li_6_PS_5_Br, Li_6_PS_5_Cl, and Li_5.5_PS_4.5_Cl_1.5_ were ruled out *via* neutron powder diffraction down to 5 K (Fig. S25–S28). A more detailed discussion on the low-temperature structure characterization can be found in the SI (Section S8).

**Fig. 7:**
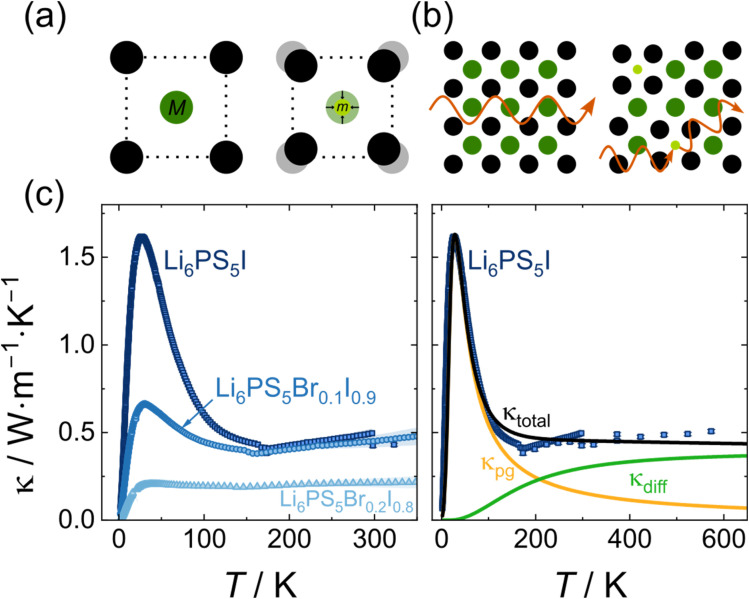
(a) The substitution of an iodide ion (dark green) with a smaller, lighter bromide ion (light green) introduces a mass and size contrast and causes the lattice to shrink locally. (b) These lattice perturbations allow phonons, depicted in orange, to exhibit enhanced phonon scattering. (c) Left panel: Influence of the loss of structural order on the thermal conductivity, drastically reducing and ultimately suppressing the phonon peak. Right panel: Fit of an analytical two-channel model to the experimental thermal conductivity of Li_6_PS_5_I. High-temperature thermal conductivity data were taken from the literature.^66^

The increasing disorder within Li_6_PS_5_Br_*x*_I_1−*x*_ may disrupt the flow of thermal energy, as the mass contrast and the radius difference between bromide and iodide ions introduce point defect scattering sites (Fig. 7a and b). Consistent with this hypothesis, a systematic reduction in thermal conductivity is observed with increasing bromine content across all measured temperatures (Fig. 7c). For both Li_6_PS_5_I and Li_6_PS_5_Br_0.1_I_0.9_ a distinct phonon peak below the phase transition can be observed. In contrast, in Li_6_PS_5_Br_0.2_I_0.8_ only a very shallow maximum is found and its thermal conductivity remains essentially temperature-independent above 50 K. At temperatures above 170 K, Li_6_PS_5_I and Li_6_PS_5_Br_0.1_I_0.9_ exhibit a slight increase in thermal conductivity, which is in line with previously reported values of Li_6_PS_5_I up to 573 K obtained *via* laser flash analysis.^66^ The complete suppression of the phonon peak upon 20% bromine substitution underscores the sensitivity of low-temperature thermal transport towards structural disorder in lithium argyrodites and potentially in solid electrolytes in general.

The pronounced difference between the impact of reduced coherence length and the effect of structural disorder on low-temperature thermal conductivity (compare Fig. 6 and 7) can be rationalized by their characteristic length scales. While the coherence length is in the order of 10^1^ nm, structural disorder acts in the range of 10^−1^ nm to 10^0^ nm. The latter provides a much higher density of defect sites for the phonon gas-type phonons to be scattered on. Consequently, only in well-ordered systems, like monoclinic Li_6_PS_5_I, are phonons able to propagate significant distances, allowing for the observation of a phonon peak.

A detailed understanding of the temperature dependence of thermal conductivity, the relative contributions of phonon gas and diffuson-like transport, the effects of liquid-like ion dynamics, anharmonic potential energy landscapes, and coherence loss is essential for rationally engineering solid electrolytes. These factors govern how thermal energy is transported through the disordered and often dynamically fluctuating ionic frameworks of such materials. Insights into these mechanisms enable the targeted tuning of lattice thermal conductivity, *e.g.*, to manage heat dissipation in commercialized applications, thereby supporting the development of solid electrolytes with optimized transport properties and their processing procedures. However, the findings of this study imply that enhancing diffuson-type transport may require fundamentally different structural engineering strategies.

## Conclusion

This work demonstrates that post-synthesis processing procedures markedly alter crystallite size and strain and thereby also coherence length. These changes have concurrent effects on ionic transport in lithium argyrodites. While for moderate mechanical energy input, an increase in ionic conductivity is found, more severe treatments reduce ionic conductivity by disrupting the percolating Li^+^ diffusion network, despite a nearly unchanged activation energy. In contrast, the effect of reduced coherence length on vibrational frequencies and thermal conductivity was found to be comparably small, suggesting that phonon mean free paths are already suppressed below the length scale of the coherence length. Instead, the effect of structural disorder is much more significant in reducing thermal conductivity through enhanced point defect scattering. The predominance of diffuson-type heat conduction, alongside a phonon gas contribution at cryogenic temperatures, was confirmed with an analytical two-channel model successfully decomposing these channels. These dominant diffuson contributions indicate high phonon linewidths and thus very anharmonic lattice dynamics. Radial frequency distributions on the lithium ion sites further support the picture of a shallow potential energy surface with strong anharmonic contributions previously reported by Ding *et al.*^14^ for Li_6_PS_5_Cl. Collectively, these findings underscore the delicate balance between structural coherence, disorder, ion dynamics, local lattice dynamics, and heat transport in solid electrolytes. They emphasize the importance of the optimization of mechanical treatment protocols to maximize ionic conductivities. In contrast, the thermal conductivity is mainly unaffected by such treatments, potentially shifting the major part of engineering temperature control in such solid-state batteries towards an external thermal management system instead of the solid electrolyte.

## Conflicts of interest

The authors declare no competing financial interest.

## Supplementary Material

TA-013-D5TA07185B-s001

## Data Availability

All data of this study are available in datastore by Universität Münster under the DOI: https://doi.org/10.17879/02978575924. Raw data of neutron scattering experiments conducted at ORNL are available under the DOI: https://doi.org/10.14461/oncat.data/2998017. Raw data of white beam normalization data used for the ARCS and POWGEN experiments are available under the DOI: https://doi.org/10.14461/oncat.data/2570733 and https://doi.org/10.14461/oncat.data/2571204, respectively. The proposal and experimental report of the beamtime conducted at D2B are available under the DOI: https://doi.org/10.5291/ILL-DATA.5-22-824. Supplementary information: computational parameters, refinements of total scattering analysis, analysis of amorphous content, Nyquist plots, Raman spectroscopy spectra, additional plots on QENS, activation energies found with NMR, additional plots on lattice dynamics, analytical two-channel model, and low-temperature X-ray and neutron diffraction. See DOI: https://doi.org/10.1039/d5ta07185b.
