# Supplementary material for: Impact of structural coherence and disorder on the ionic transport and lattice dynamics in Li+-conducting argyrodites
Source: J Mater Chem A Mater. 2025 Oct 7;13(45):39211–28. doi: 10.1039/d5ta07185b (PMC12541574; doi:10.1039/d5ta07185b)
Supplement: TA-013-D5TA07185B-s001 [file TA-013-D5TA07185B-s001.pdf]

Supplementary Information (SI) for Journal of Materials Chemistry A.
This journal is © The Royal Society of Chemistry 2025

Supporting Information – Impact of Structural Coherence and Disorder on the Ionic Transport and Lattice Dynamics in Li^+ - conducting Argyrodites

Thorben Böger[a,b], Kyra Strotmann[a], Vasiliki Faka[a], Oliver Maus[a,b], Douglas L. Abernathy[c], Garrett E. Granroth[c], Niina H. Jalarvo[c], Cheng Li[c], Emmanuelle Suard[d], Wolfgang G. Zeier*[a,b,e]

[a]*Institute of Inorganic and Analytical Chemistry, University of Münster, D-48149 Münster, Germany*

[b]*International Graduate School for Battery Chemistry, Characterization, Analysis, Recycling and Application (BACCARA), University of Münster, D-48149 Münster, Germany*

[c]*Neutron Scattering Division, Oak Ridge National Laboratory, Oak Ridge, 37831, TN, United States*

[d]*Diffraction group, Institute Laue-Langevin (ILL), 71 avenue des Martyrs, 38000 Grenoble, France*

[e]*Institute of Energy Materials and Devices (IMD), IMD-4: Helmholtz-Institut Münster, Forschungszentrum Jülich, 48149 Münster, Germany*

Corresponding author emails: wzeier@uni-muenster.de

S1: Computational parameters

Table S1. Computational parameters used during the relaxation runs.

Computational parameter	***Relaxation***
Plane wave basis set cut-off energy / eV	650
k-points mesh	*Γ*-centered 6×6×6 (corresponding to a density of ≈5.4 Å)
Total energy convergence criterion / eV	10^{-8} (corresponding to < 10^{-9} eV/atom)
Force convergence criterion / eV·Å^{-1}	10^{-3}

Table S2. Lattice vectors of conventional and primitive unit cell found from experimental characterization and computational relaxation.

Material	a_{exp} / Å		a_{calc} / Å
	conventional u. c.	***primitive u. c.***	***primitive u. c.***
Li_6PS_5Cl	9.8376	6.9562	6.9483
Li_6PS_5Br	9.9917	7.0652	7.0180

Table S3. Computational parameters used during the force calculation runs and phonopy processing.

Computational parameter	***Force calculation***
Super cell size	2×2×2 (containing 104 atoms)
Displacement distance / Å	0.01
Plane wave basis set cut-off energy / eV	650
k-points mesh	*Γ*-centered 3×3×3 (corresponding to a density of ≈5.4 Å)
Total energy convergence criterion / eV	10^{-8} (corresponding to < 10^{-10} eV/atom)
q-mesh	18×18×18

The k-point density ρ_k was calculated from the number of k-points N_k and the reciprocal unit cell vector b.

$$\rho_k = \frac{N}{2\pi \cdot b} \tag{S1}$$

S2: X-ray scattering

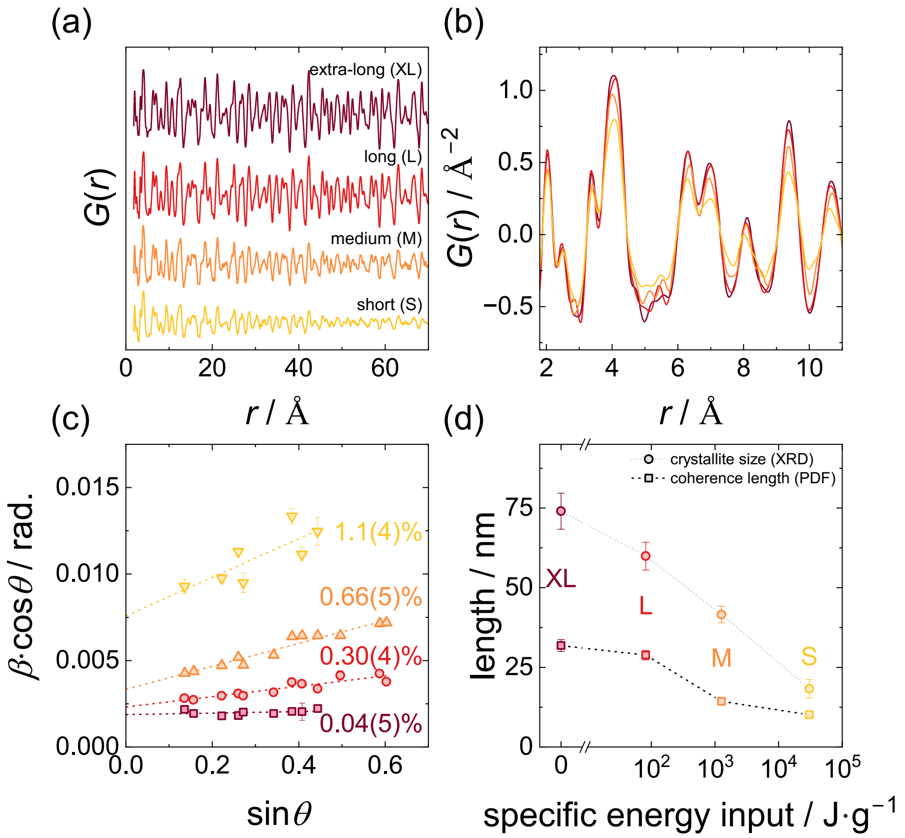

Figure S1. (a) Long- and (b) short-range PDF of $Li_{5.5}PS_{4.5}Cl_{1.5}$ for the different post-synthesis processing methods indicating the amount of disorder in the local structure and the loss in coherence. The peaks at the lowest distances of 2.1 Å and 3.4 Å can be assigned to P-S and S-S bonds of the PS_4^{3-} units, respectively. (c) Williamson-Hall analysis of $Li_{5.5}PS_{4.5}Cl_{1.5}$ with increasing amounts of strain for harsher mechanical input. (d) Coherence length as obtained from PDF and the Williamson-Hall analysis. Although both methods differ of up to a factor of 2, the general trend and order of magnitude agree for both methods. An analogous plot for Li_6PS_5Br is given in Figure 2 of the main text.

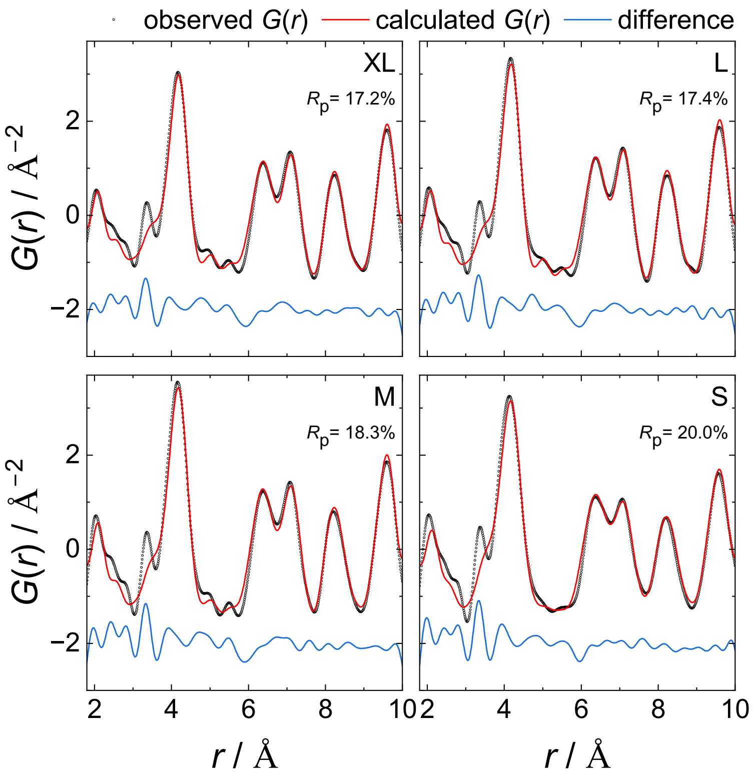

Figure S2. Observed and calculated reduced pair-distribution functions for the different post-synthesis processing methods of Li_6PS_5Br. The refinement was conducted with a r_{max}=10 Å. For visual clarity the difference is offset by −2 $Å^{-2}$.

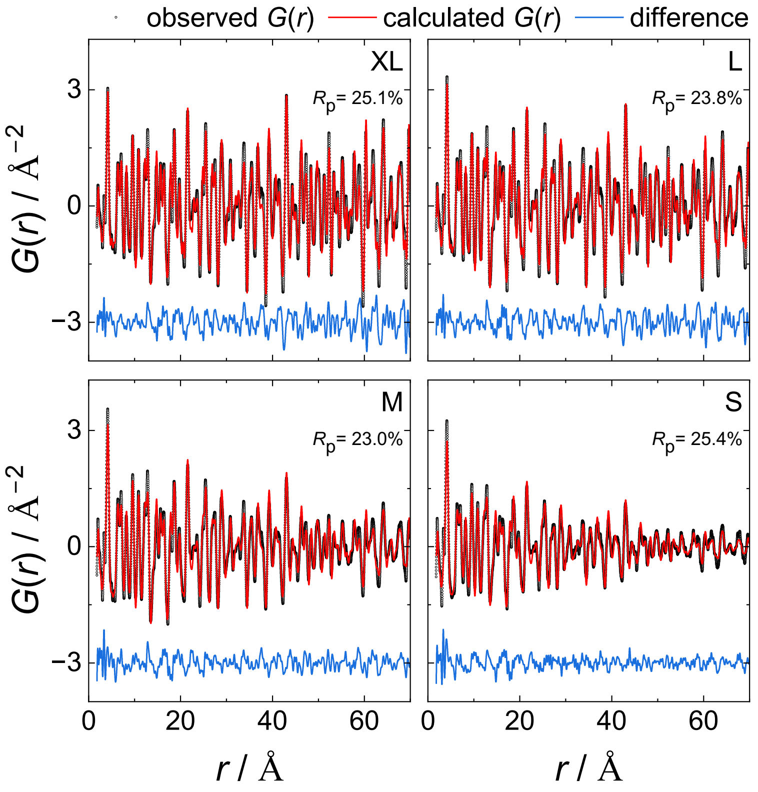

Figure S3. Observed and calculated reduced pair-distribution functions for the different post-synthesis processing methods of Li_6PS_5Br. The refinement was conducted with a r_{max}=70 Å. For visual clarity the difference is offset by −3 $Å^{-2}$.

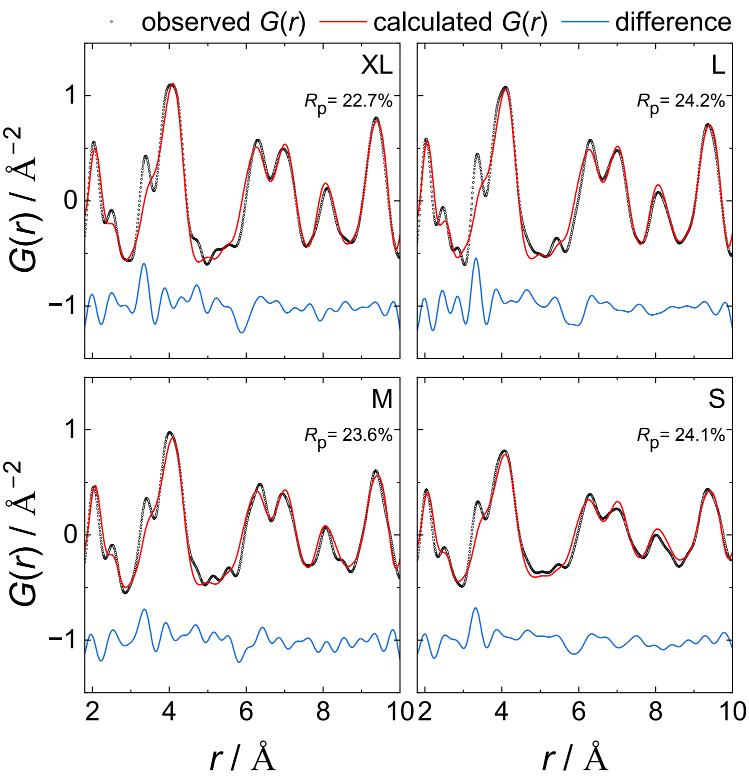

Figure S4. Observed and calculated reduced pair-distribution functions for the different post-synthesis processing methods of $Li_{5.5}PS_{4.5}Cl_{1.5}$. The refinement was conducted with a r_{max}=10 Å. For visual clarity the difference is offset by −1 $Å^{-2}$.

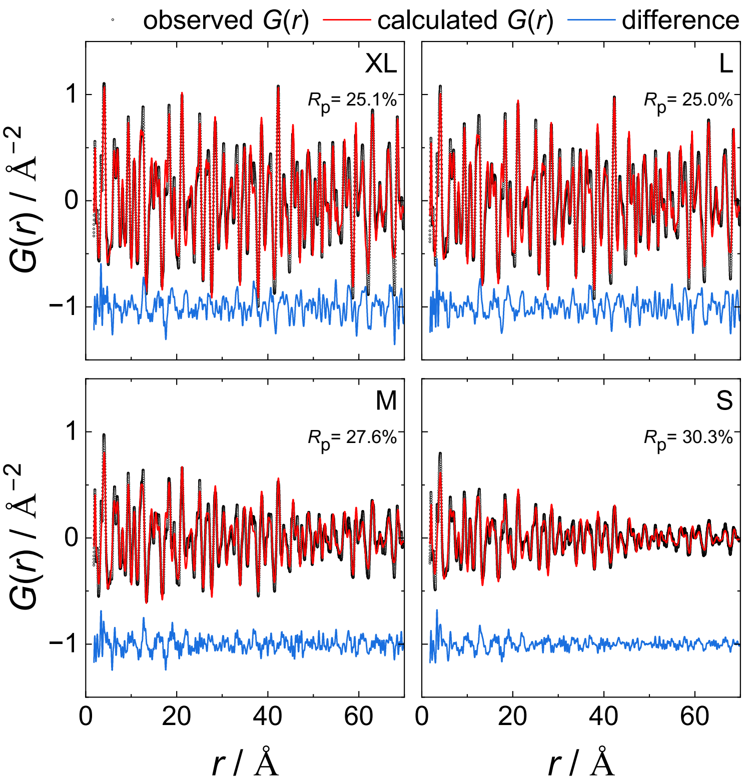

Figure S5. Observed and calculated reduced pair-distribution functions for the different post-synthesis processing methods of $Li_{5.5}PS_{4.5}Cl_{1.5}$. The refinement was conducted with a r_{max}=70 Å. For visual clarity the difference is offset by −1 $Å^{-2}$.

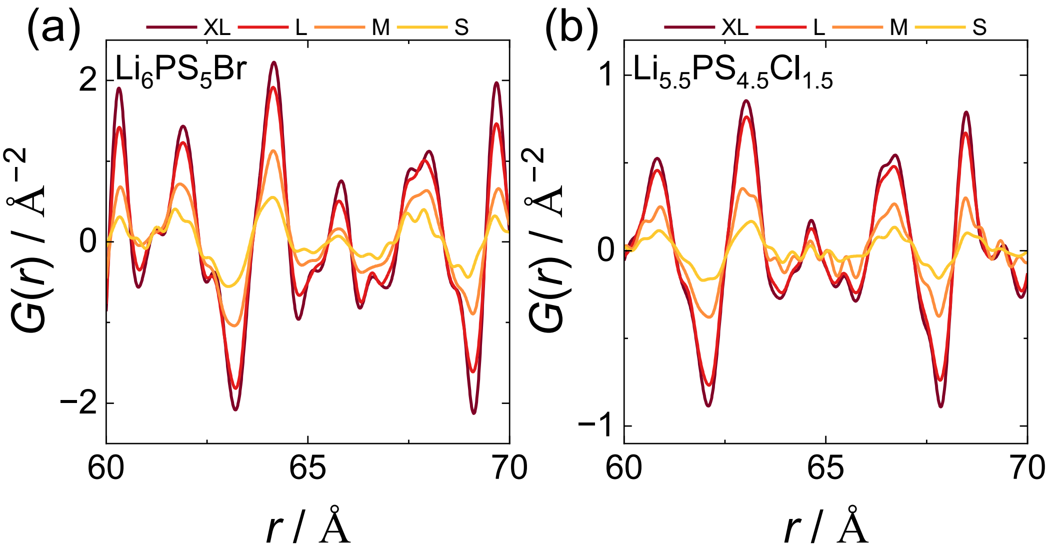

Figure S6. G(r) of (a) Li_6PS_5Br and (b) $Li_{5.5}PS_{4.5}Cl_{1.5}$ at high distances demonstrating broader peaks and less intense signals for shorter coherence lengths (higher mechanical input).

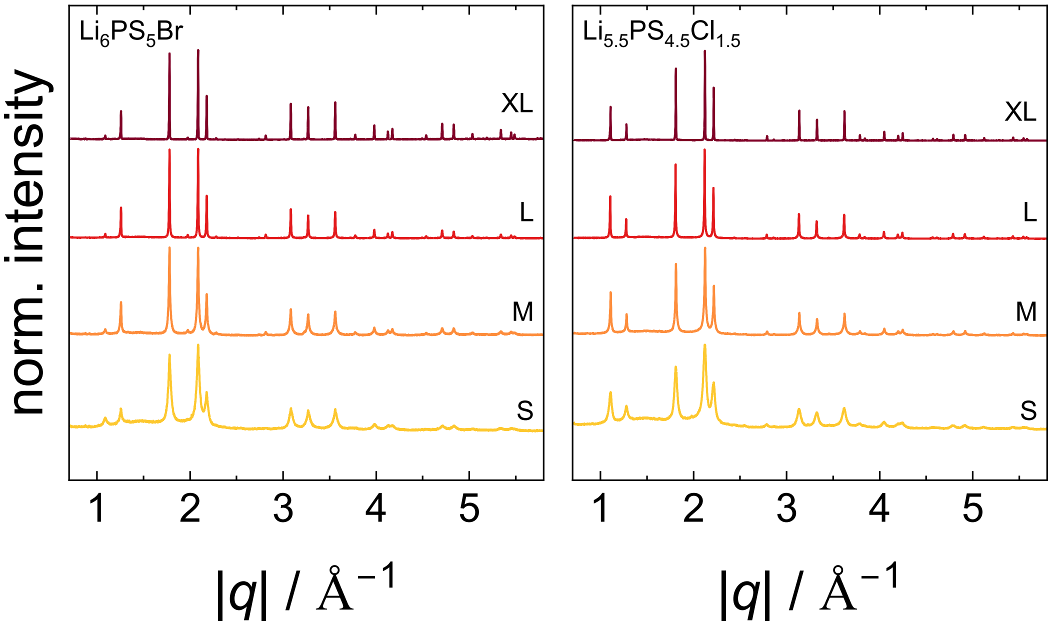

Figure S7. XRD patterns of Li_6PS_5Br and $Li_{5.5}PS_{4.5}Cl_{1.5}$ after the different post-synthesis treatments. With increasing harshness of the processing and reduced coherence length, a clear trend towards larger peak broadening can be noticed.

Table S4. Actual and refined Li_6PS_5Br (LPSBr) contents of LPSBr-Si composite used to determine the amorphous fractions of amorphous LPSBr.

Sample by coherence length	***w_{actual}(LPSBr) in %***	***$w_{refined}$(LPSBr) in %***	***w(am. LPSBr) in %***
extra-long (XL)	78.7(4)	82.3(10)	−4.6(14)
long (L)	80.0(4)	82.0(14)	−2.5(18)
medium (M)	80.5(3)	79.1(5)	1.8(7)
short (S)	80.6(3)	77.4(7)	3.9(9)

Table S5. Actual and refined $Li_{5.5}PS_{4.5}Cl_{1.5}$ (LPSCl1.5) contents of LPSCl1.5-Si composite used to determine the amorphous fractions of amorphous LPSCl1.5.

Sample by coherence length	***w_{actual}(LPSCl1.5) in %***	***$w_{refined}$(LPSCl1.5) in %***	***w(am. LPSCl1.5) in %***
extra-long (XL)	80.1(3)	83.3(12)	−4.0(15)
long (L)	79.7(3)	80(2)	−1(3)
medium (M)	80.4(3)	81.0(5)	−0.5(8)
short (S)	81.2(3)	82.3(4)	−1.3(7)

S3: Impedance spectroscopy

Table S6. Relative densities $\rho_{rel.}$ and cell constants K of pellets used in the impedance spectroscopy to characterize ionic transport. The relative density was obtained by dividing the geometrically measured density by the crystallographic density. The cell constants were calculated as ratio of pellet thickness to electrode area.

Sample by coherence length	***Li_6PS_5Br***		***$Li_{5.5}PS_{4.5}Cl_{1.5}$***	
	$\rho_{rel.}$	***K / cm^{-1}***	***$\rho_{rel.}$***	***K / cm^{-1}***
extra-long (XL)	87%	0.495	88%	0.706
long (L)	86%	0.509	87%	0.529
medium (M)	85%	1.029	83%	0.808
short (S)	85%	0.811	85%	0.802

To fit the impedance responses, a (RP)-P equivalent circuit (Figure S8a) was used. If the data quality or the amount of data points of the depressed semicircle, modelled by

the (RP) circuit was not sufficient for a stable fit, only the tail of the impedance response was fitted and the (RP) circuit was replaced by a Ohmic resistor (Figure S8b).

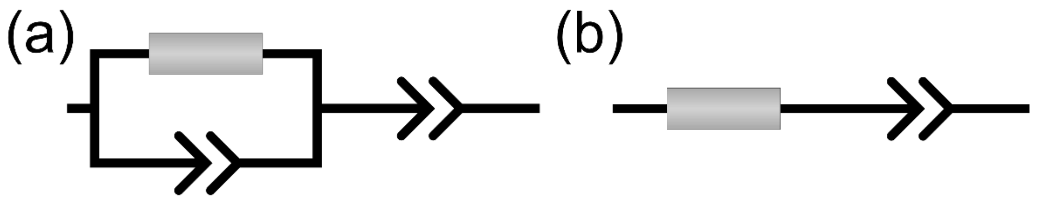

Figure S8. Equivalent circuits used for fitting impedance responses. Either a (a) (RP)-P or (b) R-P circuit was used. Latter was only employed, if to little datapoints were available to fit the depressed semicircle with a (RP) circuit.

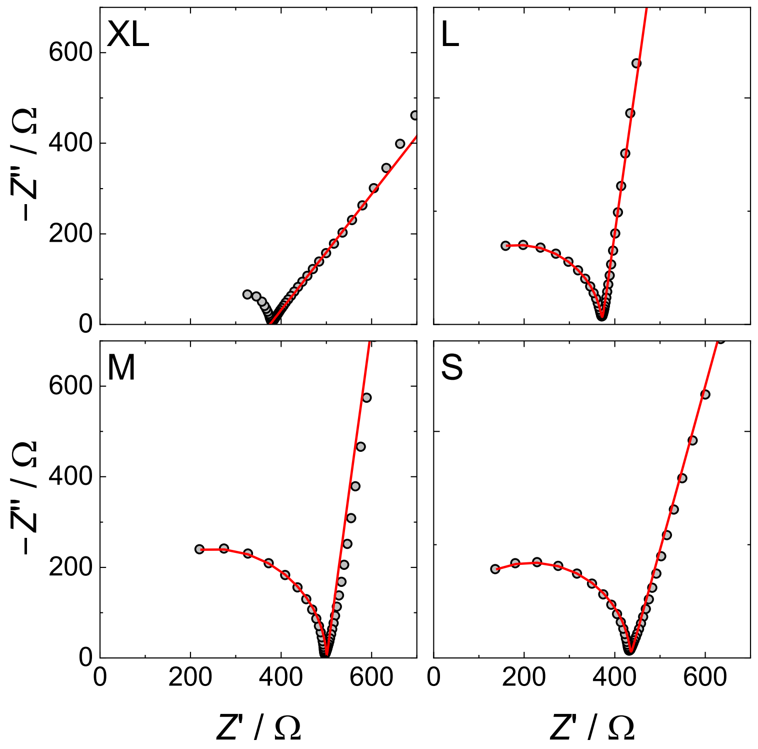

Figure S9. Nyquist plot and corresponding fits of impedance responses of Li_6PS_5Br with different coherence lengths at 298 K.

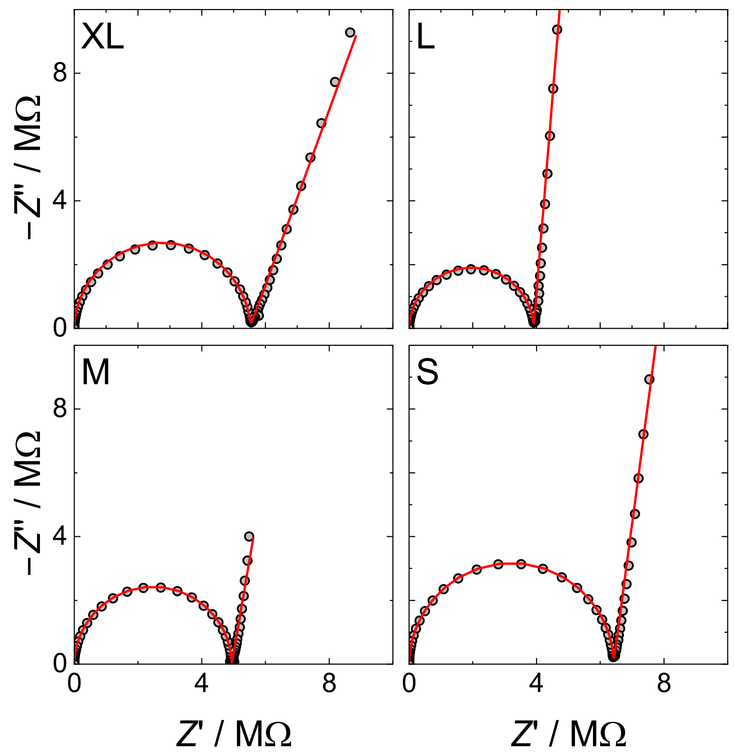

Figure S10. Nyquist plot and corresponding fits of impedance responses of Li_6PS_5Br processed with different coherence lengths at 173 K.

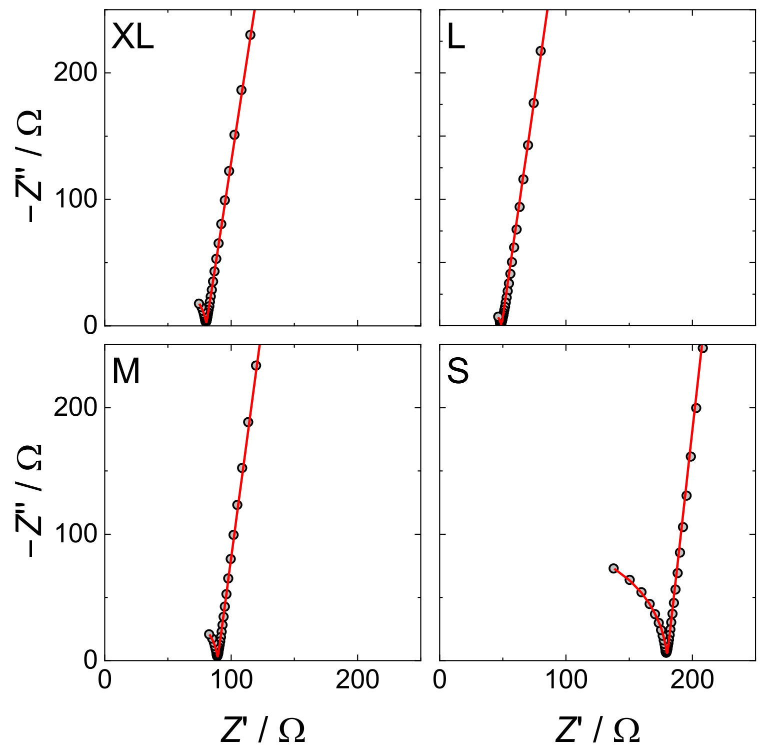

Figure S11. Nyquist plot and corresponding fits of impedance responses of $Li_{5.5}PS_{4.5}Cl_{1.5}$ with different coherence lengths input at 298 K.

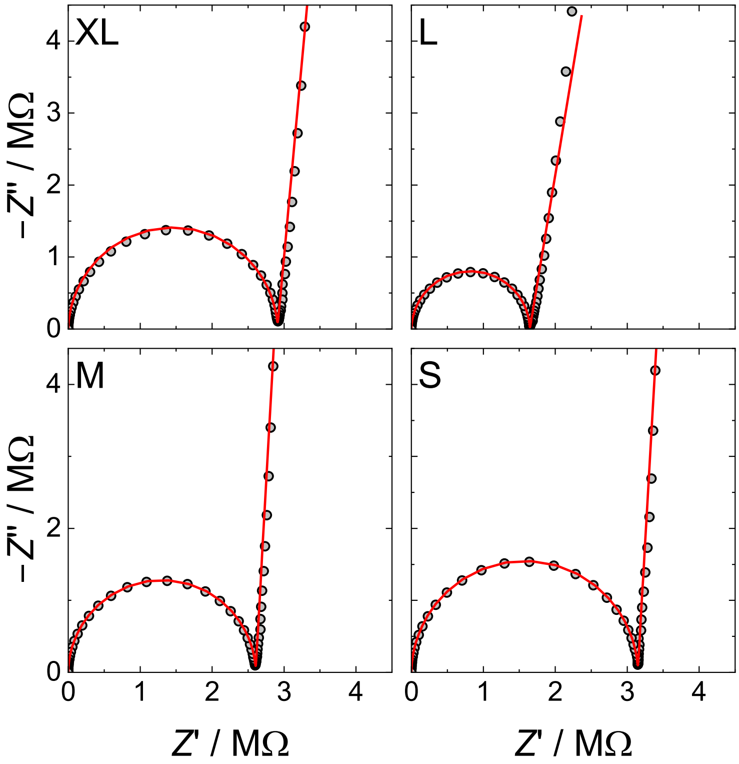

Figure S12. Nyquist plot and corresponding fits of impedance responses of $Li_{5.5}PS_{4.5}Cl_{1.5}$ with different coherence lengths at 173 K.

S4: Raman spectroscopy

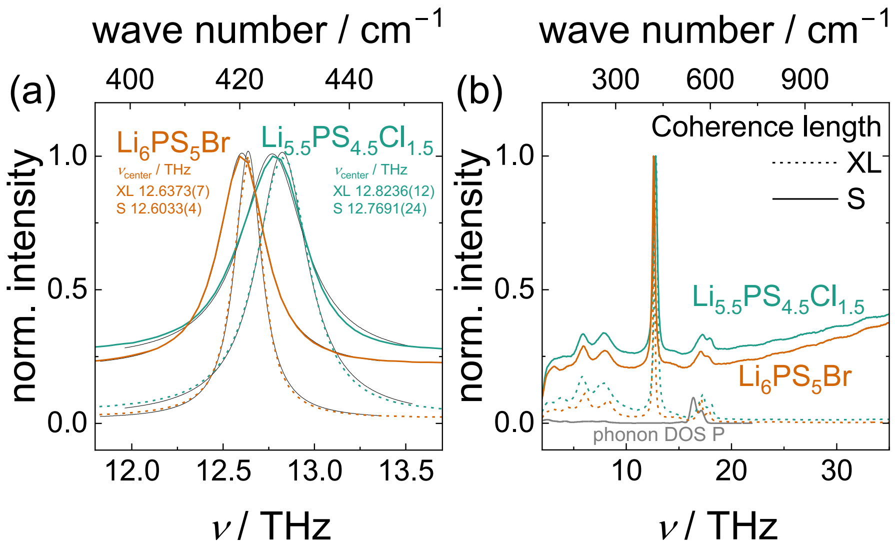

Figure S13. Raman spectra of samples with lowest coherence ("S") and highest coherence ("XL"). The zoomed in panel (a) depicts a slight red-shift for the low-coherence samples. Thin black lines represent Lorentzian fits of the peaks. The obtained center frequencies with their respective uncertainties are given in the plot. Although the fits tend to overestimate the high frequency and underestimate the low frequency flank of the peak for $Li_{5.5}PS_{4.5}Cl_{1.5}$, the offer greatly enhanced accuracy over

the instrument resolution. The entire spectrum in (b) shows good agreement between calculated energies of phosphorous vibration and the experimentally found frequencies.

S5: Quasi-elastic neutron scattering

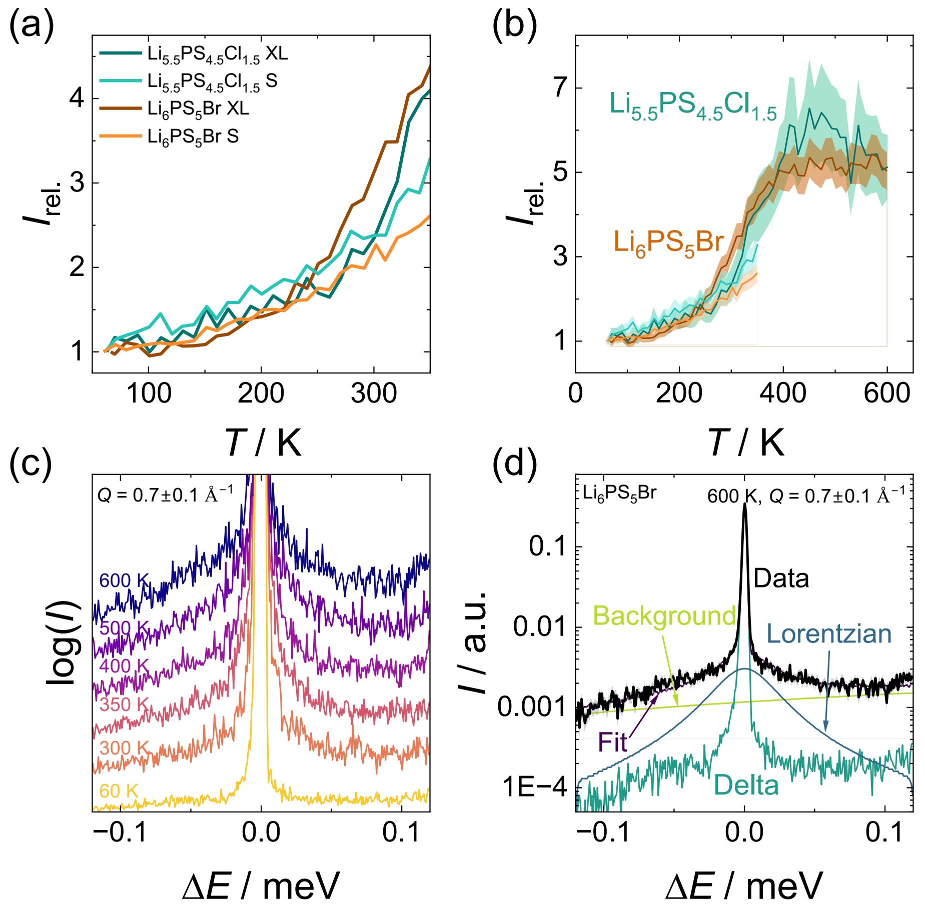

Figure S14. (a) and (b) Relative intensity of quasi-elastically scattered neutrons integrated over the entire Q-range and -120 µeV to -10 µeV and 10 µeV to 120 µeV, and normalized to the lowest temperature. The increase in relative intensity below the onset of quasi-elastic scattering at 250–300 K is caused by Debye-Waller behavior of the inelastic background. For visual clarity, uncertainties are not shown in (a). "XL" and "S" refer to the coherence length of each sample. (c) Stacked plot of the measured intensity of pristine Li_6PS_5Br with respect to energy transfer at Q=0.7±0.1 Å at various temperatures. With increasing temperature, the quasi-elastic broadening becomes more apparent. (d) Exemplary fit of a QENS signal (here Li_6PS_5Br, 600 K, Q=0.7±0.1 $Å^{-1}$) using Equation (2) of the main text.

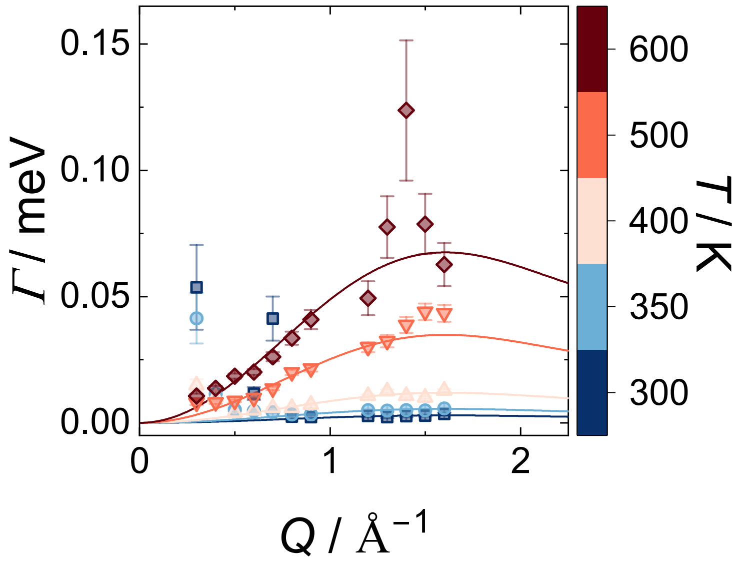

Figure S15. Q-dependence of the Lorentzian linewidth at various temperatures for $Li_{5.5}PS_{4.5}Cl_{1.5}$ and corresponding fits using the Chudley-Elliott model with fixed jump length. Q bins with considerable contributions by Bragg peaks were excluded and are not shown.

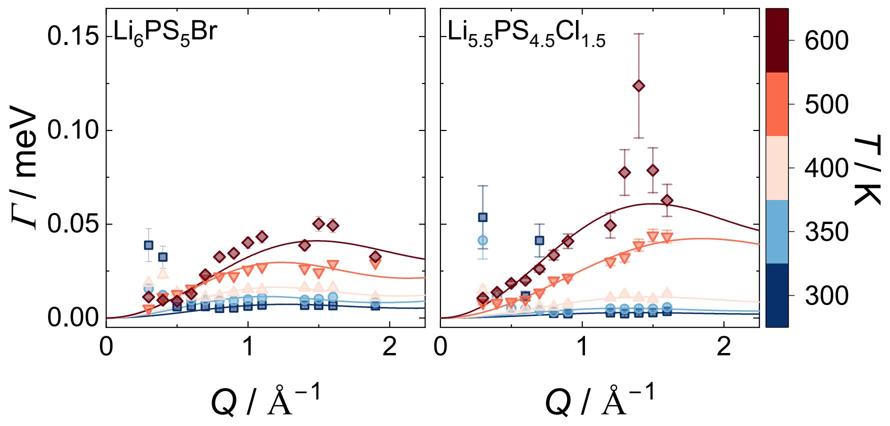

Figure S16. Q-dependence of the Lorentzian linewidth at various temperatures for Li_6PS_5Br and $Li_{5.5}PS_{4.5}Cl_{1.5}$ with corresponding fits using the Chudley-Elliott model. The jump length was not fixed. Q bins with considerable contributions by Bragg peaks were excluded and are not shown.

Table S7. Jump lengths d, mean residence times τ, and corresponding diffusion coefficients D obtained from Chudley-Elliott fits of the linewidth in Li_6PS_5Br.

**Material**	**Temperature / K**	***d* / Å**	***τ* / ps**	***D* / 10^{-10} m²·s⁻¹**
**Li_6PS_5Br**	300	3.5(2)	109(6)	1.9(3)
**Li_6PS_5Br**	350	4.0(2)	70(3)	3.8(5)
**Li_6PS_5Br**	400	3.8(2)	49(2)	4.8(6)
**Li_6PS_5Br**	500	3.63(16)	27.0(12)	8.1(8)

**Li_6PS_5Br**	600	3.02(20)	19.5(17)	7.8(12)

Table S8. Jump lengths d, mean residence times τ, and corresponding diffusion coefficients D obtained from Chudley-Elliott fits of the linewidth in $Li_{5.5}PS_{4.5}Cl_{1.5}$.

Material	Temperature / K	*d* / Å	*τ* / ps	*D* / 10^{-10} $m^2 \cdot s^{-1}$
**$Li_{5.5}PS_{4.5}Cl_{1.5}$**	300	3.2(12)	$2.8(5) \cdot 10^2$	0.6(5)
**$Li_{5.5}PS_{4.5}Cl_{1.5}$**	350	3.5(4)	153(13)	1.3(3)
**$Li_{5.5}PS_{4.5}Cl_{1.5}$**	400	3.0(3)	71(6)	2.2(4)
**$Li_{5.5}PS_{4.5}Cl_{1.5}$**	500	2.4(3)	19(3)	5.2(15)
**$Li_{5.5}PS_{4.5}Cl_{1.5}$**	600	3.0(4)	13(2)	11(3)

S5: Activation energies reported by nuclear magnetic spectroscopy experiments

Table S9. Activation energies of Li^+ diffusion in Li_6PS_5Br reported by different nuclear magnetic spectroscopy (NMR) methods. SLR stands for spin-lattice relaxation and SFG for static-field gradient.

Material	NMR method	Activation energy / eV	Reference
**Li_6PS_5Br**	^7Li SLR $T_{1\rho}$ (low T)	0.08(2)	1
**Li_6PS_5Br**	^7Li SLR $T_{1\rho}$ (low T)	0.083	2
**Li_6PS_5Br**	^7Li SLR T_1 (low T)	0.10(9)	3
**Li_6PS_5Br**	^7Li SLR T_1 (low T)	0.10	2
**Li_6PS_5Br**	^7Li SLR T_1 (high T)	0.105	4
**Li_6PS_5Br**	^7Li SLR T_1 (high T)	0.15(0)	3
**Li_6PS_5Br**	^7Li SLR $T_{1\rho}$ (high T)	0.20	2
**Li_6PS_5Br**	^7Li SLR $T_{1\rho}$ (high T)	0.20	1
**Li_6PS_5Br**	^7Li SLR T_1	0.20(1)	1
**Li_6PS_5Br**	^6Li SLR T_1	0.20(3)	1
**Li_6PS_5Br**	^7Li SLR T_1 (high T)	0.21	2
**Li_6PS_5Br**	^7Li SLR $T_{1\rho}$ (high T)	0.34(9)	3

Table S10. Activation energies of Li^+ diffusion in $Li_{5.5}PS_{4.5}Cl_{1.5}$ reported by different nuclear magnetic spectroscopy (NMR) methods. SLR stands for spin-lattice relaxation, PFG for pulsed-field gradient, and SFG for static-field gradient.

Material	NMR method	Activation energy / eV	Reference
**$Li_{5.3}PS_{4.3}Cl_{1.7}$**	^{7}Li SLR T_1 (low T)	0.081(3)	5
**$Li_{5.5}PS_{4.5}Cl_{1.5}$**	^{7}Li SLR T_1 (low T)	0.109	6
**$Li_{5.5}PS_{4.5}Cl_{1.5}$**	^{7}Li SLR T_1 (low T)	0.12	7
**$Li_{5.5}PS_{4.5}Cl_{1.5}$**	^{7}Li SLR T_1 (high T)	0.162	6
**$Li_{5.5}PS_{4.5}Cl_{1.5}$**	^{7}Li SLR T_1 (high T)	0.29	7
**$Li_{5.5}PS_{4.5}Cl_{1.5}$**	^{7}Li PFG	0.29(1)	8
**$Li_{5.5}PS_{4.5}Cl_{1.5}$**	^{7}Li SFG	0.34	9
**$Li_{5.5}PS_{4.5}Cl_{1.5}$**	^{7}Li SLR T_1	0.40	9

S6: Lattice dynamics

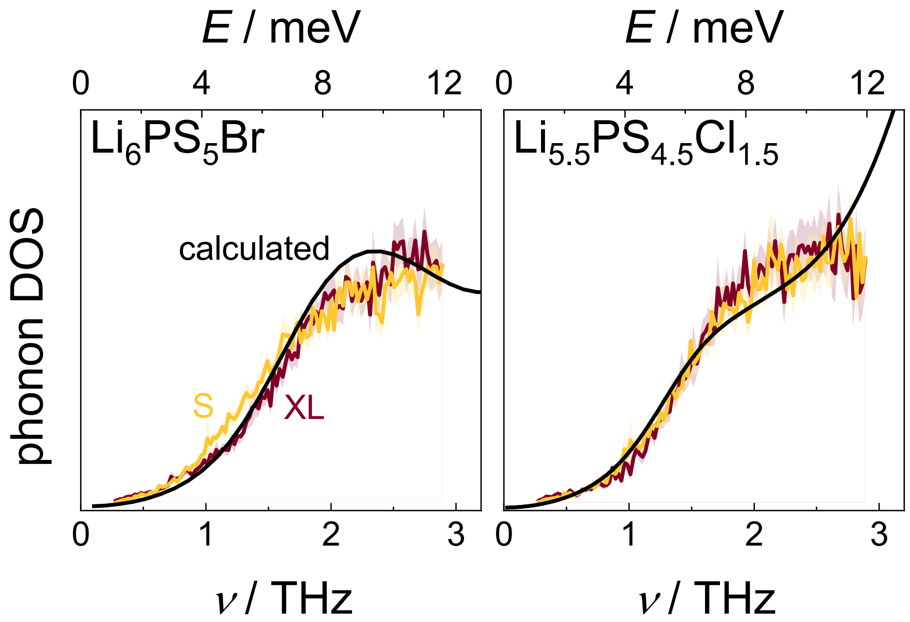

Figure S17. Phonon DOS of Li_6PS_5Br (left panel) and $Li_{5.5}PS_{4.5}Cl_{1.5}$ (right panel) at 100 K as obtained from inelastic neutron diffraction experiments using neutrons with 15 meV incident neutron energy as well as the modelled neutron-weighted phonon DOS. "XL" and "S" denote the coherence length of the respective sample.

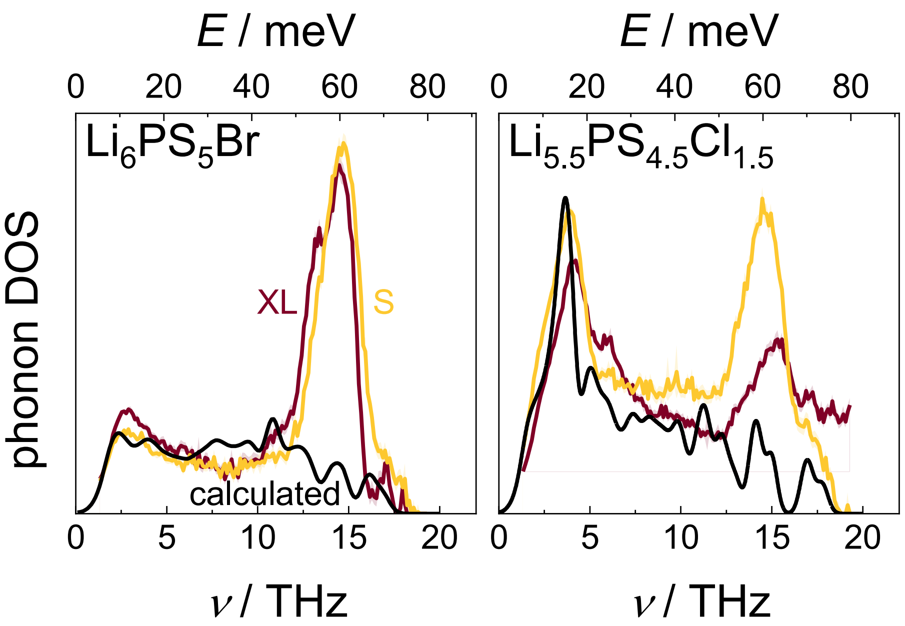

Figure S18. Phonon DOS of Li_6PS_5Br (left panel) and $Li_{5.5}PS_{4.5}Cl_{1.5}$ (right panel) at 100 K as obtained from inelastic neutron diffraction experiments using neutrons with 100 meV incident neutron energy as well as the modelled neutron-weighted phonon DOS. "XL" and "S" denote the coherence length of the respective sample.

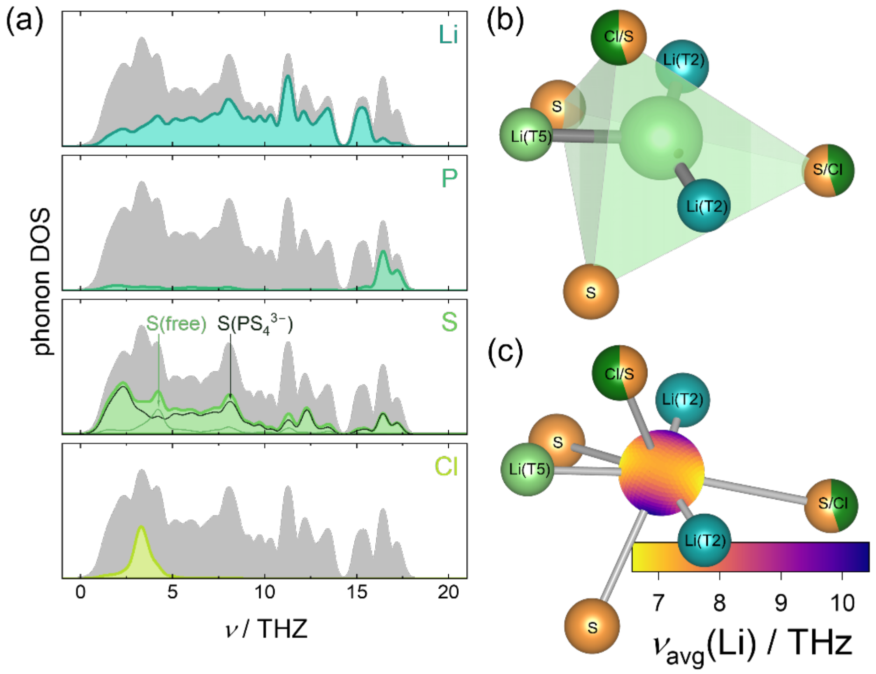

Figure S19. (a) Calculated atom-projected phonon density of states of Li_6PS_5Cl. The gray area represents the total phonon DOS. (b) Coordination environment of the T5 site in Li_6PS_5Cl. (c) Distribution of average frequencies in all spatial direction. The orientation is the same as in panel (b).

S7: Analytical two channel model of thermal conductivity

The analytical two channel model employed here has been derived by Bernges et al.[10] and used for a study on silver argyrodites. For a more detailed derivation the reader is referred to this study.

Previous studies have found that low frequency vibrations, especially acoustic phonon modes, contribute mainly to the phonon-gas type transport, whereas vibrations of higher frequency conduct thermal energy rather diffuson-like.[11–13] Although this cross-over is not sharp, the model employs a cut-off frequency, the so-called Ioffe-Regel limit, which splits the vibrational spectrum into two parts. Each part is assumed to behave fully phonon-gas like or fully diffuson-like, respectively. As diffuson thermal conductivity a formula derived by Agne et al.[14] is used. With that the thermal conductivity by diffusons κ_{diff} is:

$$\kappa_{\mathrm{diff}}(T) = \frac{n^{\frac{1}{3}} k_{\mathrm{B}}}{\pi} \int_{\nu_{\mathrm{IR}}}^{\infty} g(\nu) \cdot C(\nu) \cdot P \cdot \nu \, \mathrm{d}\nu \tag{S2}$$

where ν denotes the frequency, ν_{IR} the Ioffe-Regel frequency (cut-off frequency), n the number density of atoms, $g(\nu)$ the normalized phonon density of states, $C(\nu)$, and P an average thermal energy exchange rate between diffuson modes. P can be seen as average probability that the energy transfer of one phonon mode to another is successful. This corresponds closely to the phonon band overlap with the neighboring phonon modes. The heat capacity of a phonon mode is given by:

$$C(\nu) = \left(\frac{h\nu}{k_{\mathrm{B}}T}\right)^2 \mathrm{e}^{\frac{h\nu}{k_{\mathrm{B}}T}} \left(\mathrm{e}^{\frac{h\nu}{k_{\mathrm{B}}T}} - 1\right)^{-2} \tag{S3}$$

This is simply the phonon energy given by $h\nu$ multiplied by the Bose-Einstein statistic differentiated with respect to the temperature.

The phonon-gas type thermal transport is based on the general formula for thermal conductivity in ideal gases and is given by:

$$\kappa_{\mathrm{pg}}(T) = \frac{n k_{\mathrm{B}}}{3} \cdot \int_{0}^{\nu_{\mathrm{IR}}} g(\nu) \cdot C(\nu) \cdot v^2 \cdot \tau(\nu) \, \mathrm{d}\nu \tag{S4}$$

The speed of sound v is taken from experiments and the phonon lifetime $\tau(\nu)$ is approximated by an analytical function. The contributions of different scattering

sources are combined by adding their reciprocal values. Here, phonon-phonon scattering (ph) and grain-boundary scattering (gb) is considered.

$$\tau(\nu)^{-1} = \tau_{\mathrm{ph}}^{-1}(\nu) + \tau_{\mathrm{gb}}^{-1}(\nu) = C_1\nu^2 T + A\nu \quad \text{(S5)}$$

C_1 and A give the magnitude of both scattering phenomena and are used as parameters fitted to the experimental data. Finally, the total thermal conductivity is the sum of the phonon-gas and diffuson channels.

$$\kappa_{\mathrm{total}} = \kappa_{\mathrm{pg}} + \kappa_{\mathrm{diff}} \quad \text{(S6)}$$

S8: Low-temperature diffraction

Low temperature X-ray diffraction experiments revealed phase transitions at varying temperature ranges in bromine-substituted Li_6PS_5I. With no bromine, the phase transition from cubic $F\bar{4}3m$ (space group no. 216) to monoclinic *Cc* (space group no. 9) occurs between 158 K and 166 K (Figure S20). As the monoclinic model is capable of mimicking the cubic structure, but has much more degrees of freedom, it will always result in a lower R_{wp} value, which is used here to assess the quality of the fit. Therefore, if R_{wp} values of both cubic and monoclinic model are similar, the structure is assumed to be cubic, as for example at 166 K in Li_6PS_5I (x=0) (Figure S20a). At 162 K an increase in the R_{wp} values for both the cubic and monoclinic model could be noticed, suggesting an ongoing phase transition. The corresponding diffractogram exhibits features of both purely monoclinic and purely cubic diffractograms (Figure S20b to d). At 158 K the R_{wp} value of the monoclinic model is reduced again and stays constant for lower temperatures, whereas a further increase is noticed for the cubic model, indicating a purely monoclinic structure at this temperature. The same trends are also observed in $Li_6PS_5Br_{0.1}I_{0.9}$ (x=0.1) and $Li_6PS_5Br_{0.2}I_{0.8}$ (x=0.2). However, the onset of the phase transition is reduced from 166 K over 150 K for x=0.1 (Figure S21) to 134 K for x=0.2 (Figure S22). Especially, for x=0.2 the phase transition occurs over a wider temperature range than in Li_6PS_5I. To elucidate if slow kinetics of the phase transition cause the stretching of the phase transition, in an additional measurement, the sample was cooled rapidly from room temperature to 110 K and subsequently multiple short measurements performed. Already the first measurement exhibited a fully monoclinic phase, with no significant differences to later measurement, demonstrating that no kinetic hinderance is involved. Thermodynamically, Gibbs phase rule forbids phase

transitions stretching over a temperature range for one component systems. Another explanation is that the crystallites exhibit a distribution Br^{-}/I^{-}-ratios, resulting in different phase transition temperatures. However, this distribution cannot be very wide as the material was heated for two weeks, leaving plenty of time for concentration gradients to relaxate.[15] Moreover, different Br^{-}/I^{-}-ratios would lead to a distribution of lattice parameters, but reflections for x=0.2 are only marginally wider than for x=0. Thus, the exact reason for the wide phase transition range remains unkown.

Refinements of $Li_6PS_5Br_{0.1}I_{0.9}$ and $Li_6PS_5Br_{0.2}I_{0.8}$ indicate only a minor degrees of Br^{-}/S^{2-} disorder. However, given the low substitution degree of at most 20% bromide and, based on the synthesis procedure, an expected degree 15% Br^{-}/S^{2-} disorder, the population of bromide ions on the free sulfide position is expected to be at most 3%. Such minor differences are close the uncertainty of the refinement.

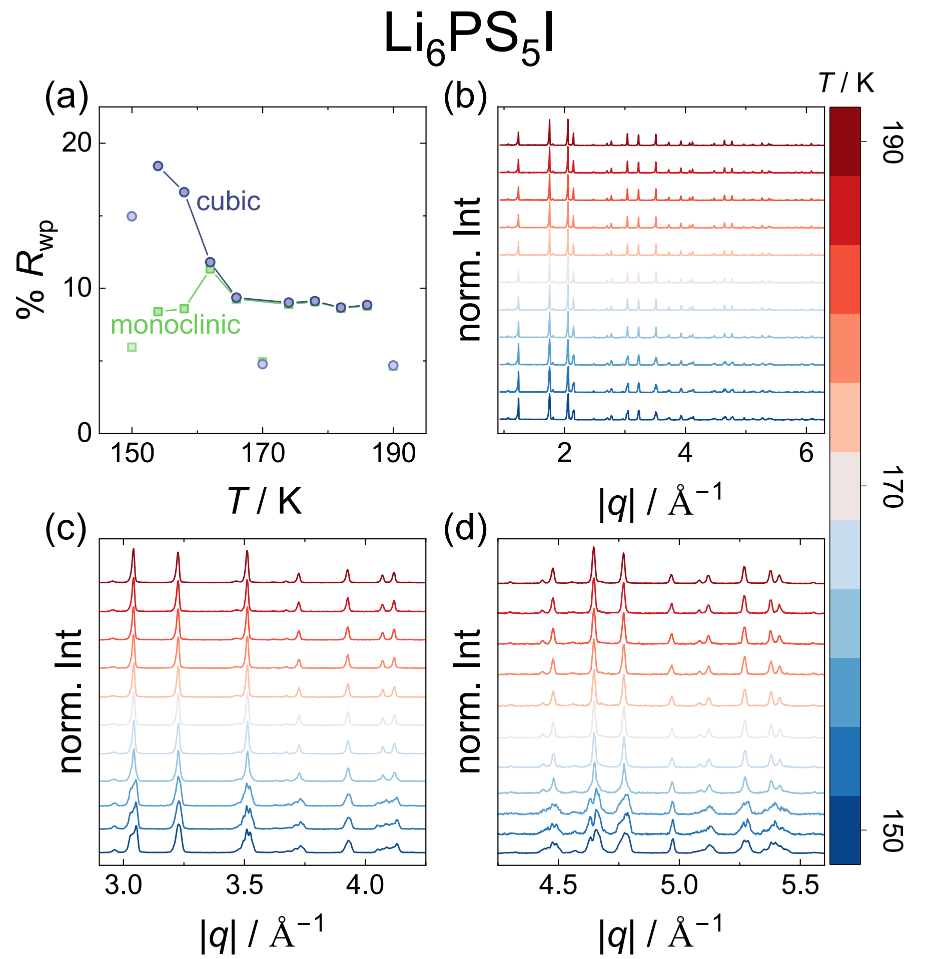

Figure S20. Low temperature XRD measurements on Li_6PS_5I. (a) R_{wp} indicating the quality of the refinement as function of temperature. The phase transition occurs between 158 K and 166 K. Lighter symbols denote measurements with prolonged acquisition time, and thus better data quality. At temperatures where no symbol of the

monoclinic model is visible, it is covered by the datapoints of the cubic model. (b) X-ray diffractograms over the entire q-range. (c) and (d) Zoom on the medium and high q-range, respectively, highlighting the changes in the diffractograms upon phase transition.

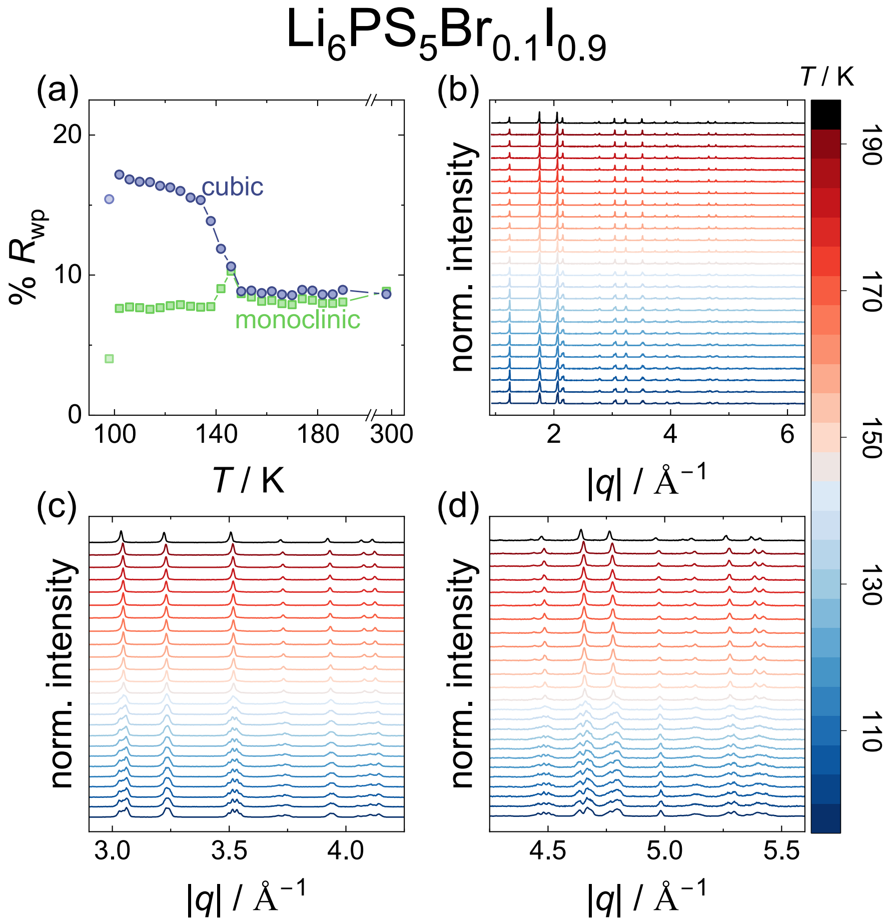

Figure S21. Low temperature XRD measurements on $Li_6PS_5Br_{0.1}I_{0.9}$. (a) R_{wp} indicating the quality of the refinement as function of temperature. The phase transition occurs between 138 K and 150 K. Lighter symbols denote measurements with prolonged acquisition time, and thus better data quality. At temperatures where no symbol of the monoclinic model is visible, it is covered by the datapoints of the cubic model. (b) X-ray diffractograms over the entire q-range. (c) and (d) Zoom on the medium and high q-range, respectively, highlighting the changes in the diffractograms upon phase transition.

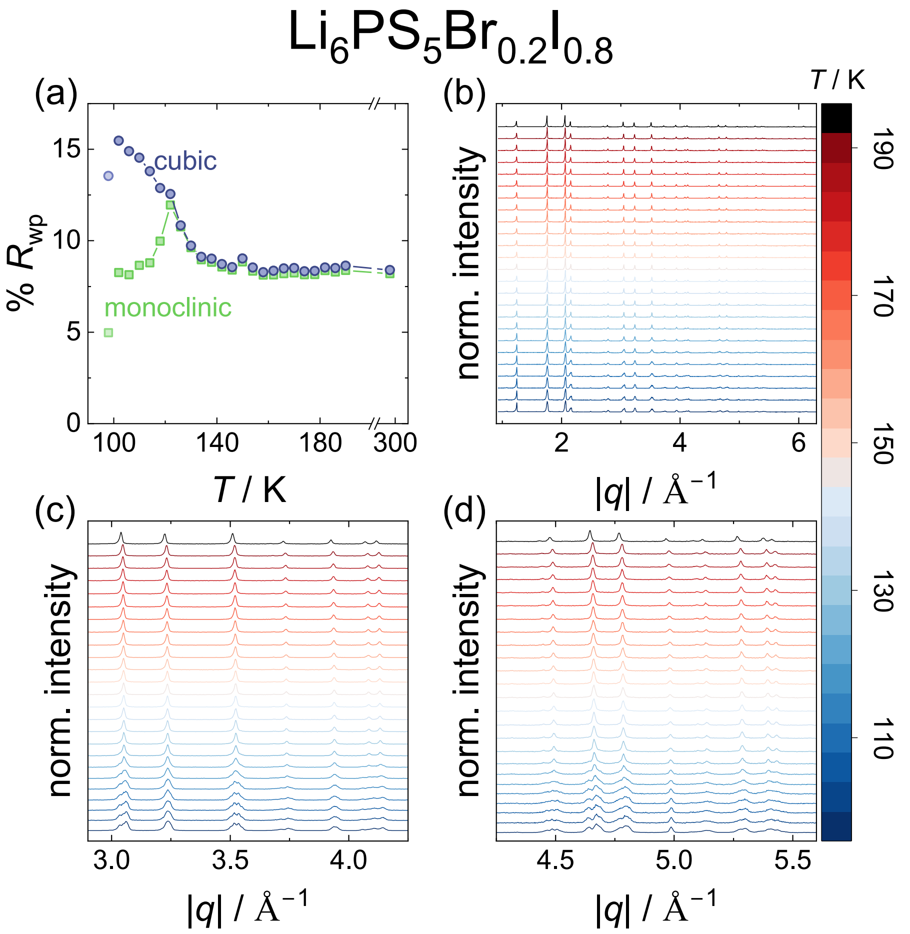

Figure S22. Low temperature XRD measurements on $Li_6PS_5Br_{0.2}I_{0.8}$. (a) R_{wp} indicating the quality of the refinement as function of temperature. The phase transition occurs between 106 K and 134 K. Lighter symbols denote measurements with prolonged acquisition time, and thus better data quality. At temperatures where no symbol of the monoclinic model is visible, it is covered by the datapoints of the cubic model. (b) X-ray diffractograms over the entire q-range. (c) and (d) Zoom on the medium and high q-range, respectively, highlighting the changes in the diffractograms upon phase transition. The diffractogram in black corresponds to measurement at room temperature.

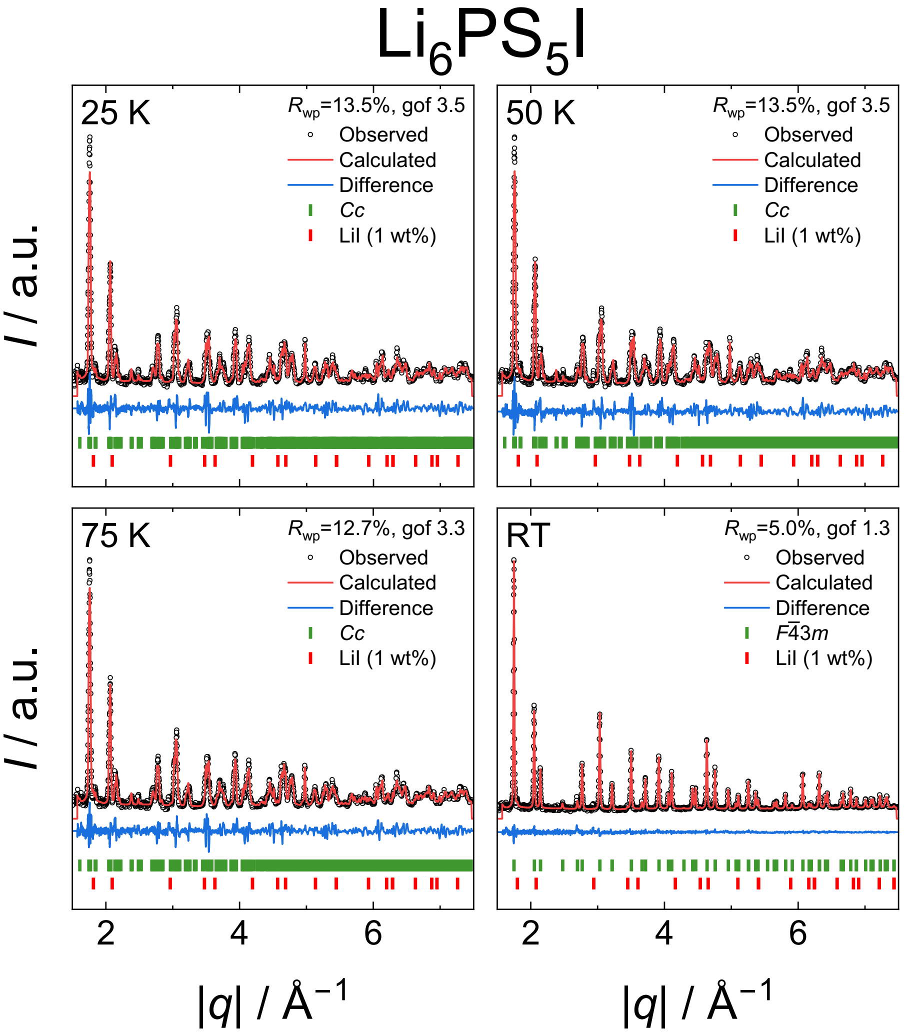

Figure S23. Rietveld refinements of neutron powder diffractograms of Li_6PS_5I at different temperatures. At 75 K and below the structure was found to be in a fully ordered, monoclinic phase (Cc, space group no. 9).

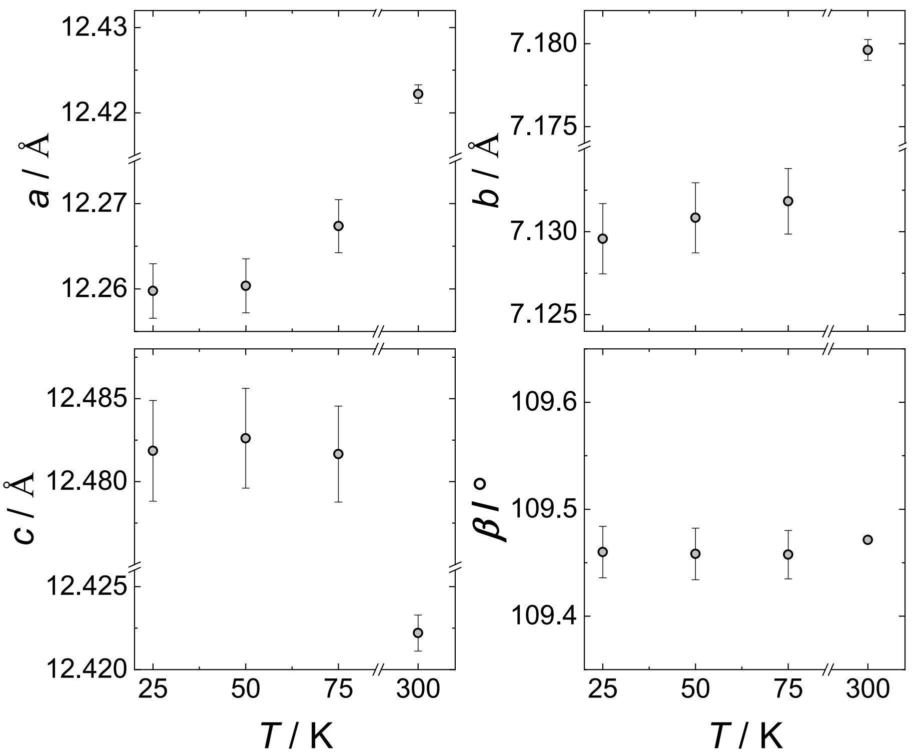

Figure S24. Evolution of the lattice parameters of monoclinic Li_6PS_5I with respect to the temperature. The lattice parameter of the cubic structure at room temperature of 10.1427(9) Å was converted to the monoclinic parameters considering the group-subgroup relationship of both space groups and the respective transformation matrix of the unit cell.

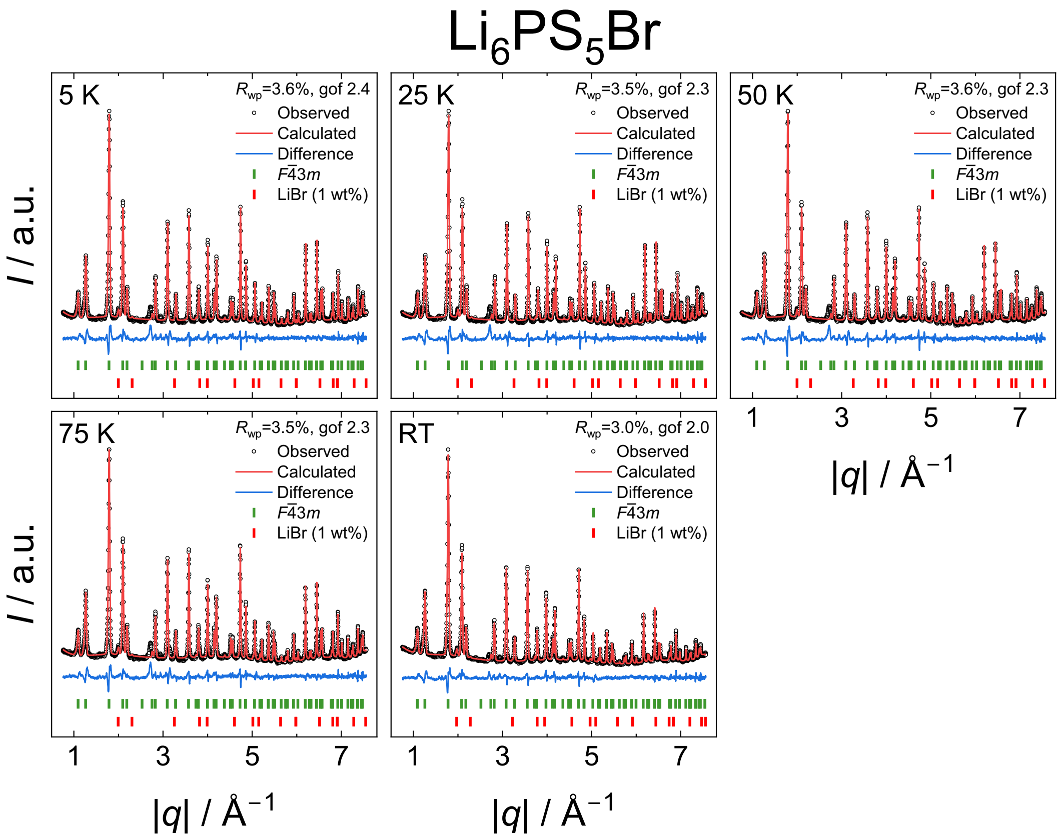

Figure S25. Rietveld refinements of neutron powder diffractograms of Li_6PS_5Br at different temperatures. No signs of a phase transition were noted.

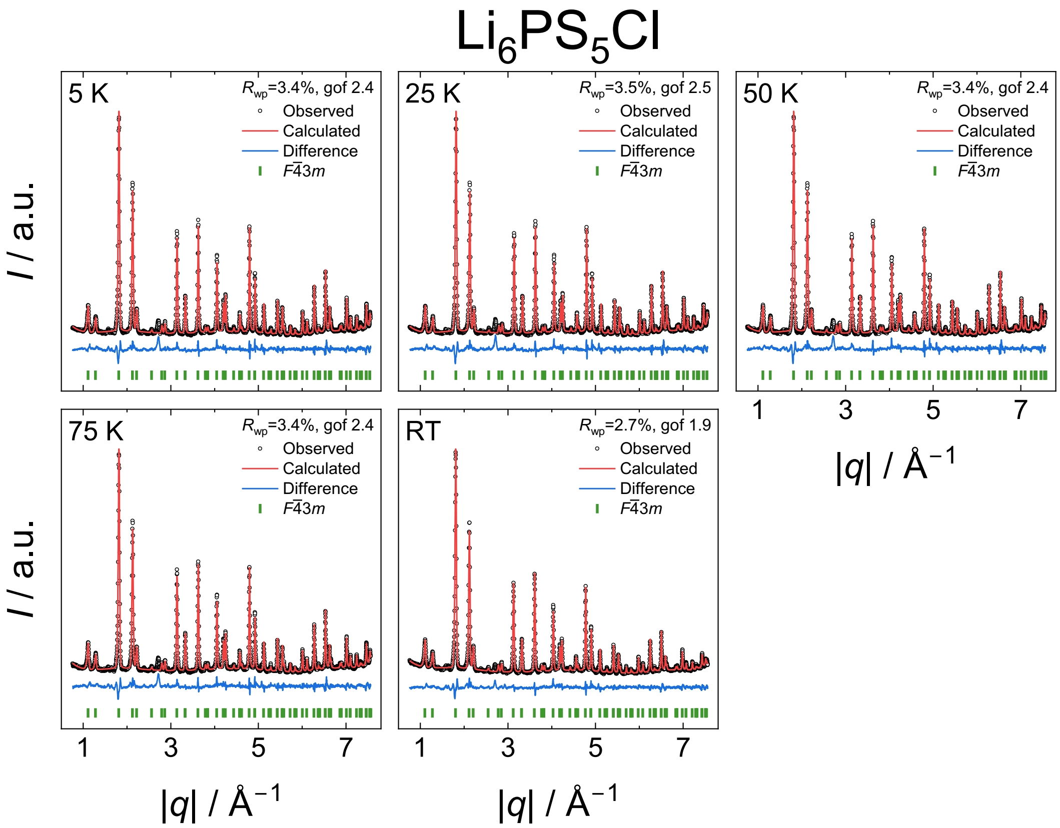

Figure S26. Rietveld refinements of neutron powder diffractograms of Li_6PS_5Cl at different temperatures. No signs of a phase transition were noted.

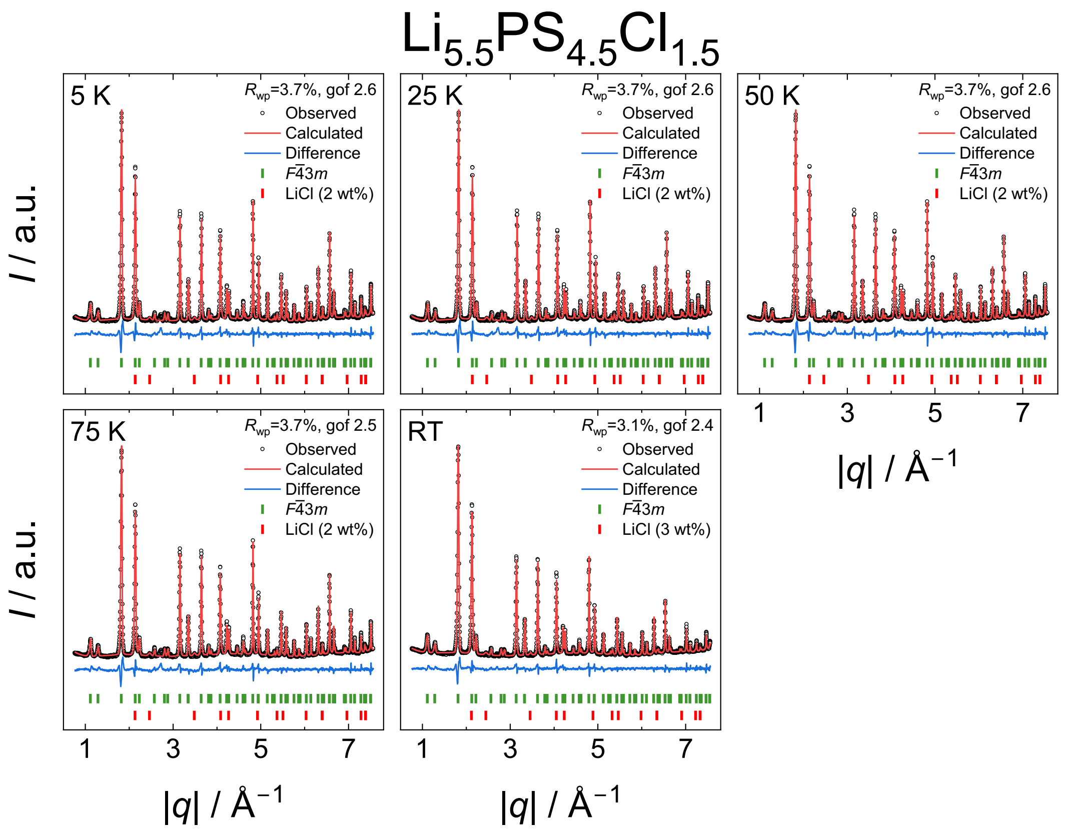

Figure S27. Rietveld refinements of neutron powder diffractograms of $Li_{5.5}PS_{4.5}Cl_{1.5}$ at different temperatures. No signs of a phase transition were noted.

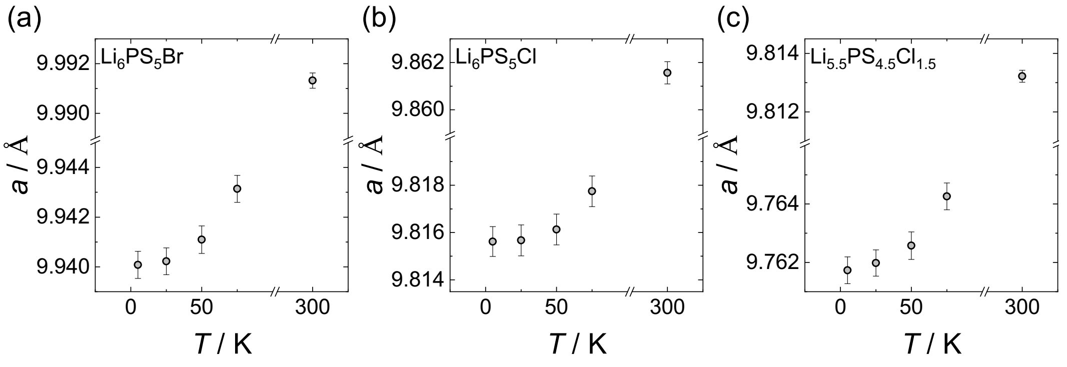

Figure S28. Evolution of the lattice parameter with respect to the temperature of (a) Li_6PS_5Br, (b) Li_6PS_5Cl, and (c) $Li_{5.5}PS_{4.5}Cl_{1.5}$.

References

(1) Epp, V.; Gün, Ö.; Deiseroth, H.-J.; Wilkening, M. Highly Mobile Ions: Low-Temperature NMR Directly Probes Extremely Fast Li+ Hopping in Argyrodite-Type Li6PS5Br. *J. Phys. Chem. Lett.* **2013**, *4* (13), 2118–2123. DOI: 10.1021/jz401003a.

(2) Hanghofer, I.; Brinek, M.; Eisbacher, S. L.; Bitschnau, B.; Volck, M.; Hennige, V.; Hanzu, I.; Rettenwander, D.; Wilkening, H. M. R. Substitutional disorder: structure and ion dynamics of the argyrodites Li6PS5Cl, Li6PS5Br and Li6PS5I. *Phys. Chem. Chem. Phys.* **2019**, *21* (16), 8489–8507. DOI: 10.1039/C9CP00664H.

(3) Yu, C.; Ganapathy, S.; van Eck, E. R. H.; van Eijck, L.; Basak, S.; Liu, Y.; Zhang, L.; Zandbergen, H. W.; Wagemaker, M. Revealing the relation between the structure, Li-ion conductivity and solid-state battery performance of the argyrodite Li 6 PS 5 Br solid electrolyte. *J. Mater. Chem. A* **2017**, *5* (40), 21178–21188. DOI: 10.1039/C7TA05031C.

(4) Yu, C.; Ganapathy, S.; van Eck, E. R. H.; Wang, H.; Basak, S.; Li, Z.; Wagemaker, M. Accessing the bottleneck in all-solid state batteries, lithium-ion transport over the solid-electrolyte-electrode interface. *Nat. Commun.* **2017**, *8* (1), 1086. DOI: 10.1038/s41467-017-01187-y. Published Online: Oct. 20, 2017.

(5) Wang, Y.; Bazak, J. D.; Zhou, L.; Zhang, Q.; Singh, B.; Nazar, L. F. Liquid-like solid-state diffusion of lithium ions in super-halide-rich argyrodite. *Cell Rep. Phys. Sci.* **2024**, *5* (12), 102314. DOI: 10.1016/j.xcrp.2024.102314.

(6) Yu, C.; Li, Y.; Willans, M.; Zhao, Y.; Adair, K. R.; Zhao, F.; Li, W.; Deng, S.; Liang, J.; Banis, M. N.; Li, R.; Huang, H.; Zhang, L.; Yang, R.; Lu, S.; Huang, Y.; Sun, X. Superionic conductivity in lithium argyrodite solid-state electrolyte by controlled Cl-doping. *Nano Energy* **2020**, *69*, 104396. DOI: 10.1016/j.nanoen.2019.104396.

(7) Maus, O.; Lange, M. A.; Frankenberg, F.; Stainer, F.; Faka, V.; Schlautmann, E.; Rosenbach, C.; Jodlbauer, A.; Schubert, J.; Janek, J.; Li, C.; Michalowski, P.; Wilkening, H. M. R.; Kwade, A.; Zeier, W. G. Influence of Post-Synthesis Processing on the Structure, Transport, and Performance of the Solid Electrolyte Li 5.5 PS 4.5 Cl 1.5 in All-Solid-State Batteries. *Adv. Energy Mater.* **2025**, *15* (5), 2403291. DOI: 10.1002/aenm.202403291. Published Online: Sep. 5, 2024.

(8) Adeli, P.; Bazak, J. D.; Park, K. H.; Kochetkov, I.; Huq, A.; Goward, G. R.; Nazar, L. F. Boosting Solid-State Diffusivity and Conductivity in Lithium Superionic Argyrodites by Halide Substitution. *Angew. Chem. Int. Ed. Engl.* **2019**, *58* (26), 8681–8686. DOI: 10.1002/anie.201814222. Published Online: May. 24, 2019.

(9) Badragheh, M. A.; Miß, V.; Ludwig, L.; Roling, B.; Vogel, M. Lithium ion dynamics and transport in the halide-rich argyrodite Li5.5PS4.5Cl1.5: Influence of heat treatment on cooperativity, heterogeneity and subdiffusion. *Solid State Ionics* **2024**, *413*, 116608. DOI: 10.1016/j.ssi.2024.116608.

(10) Bernges, T.; Peterlechner, M.; Wilde, G.; Agne, M. T.; Zeier, W. G. Analytical model for two-channel phonon transport engineering. *Mater. Today Phys.* **2023**, *35*, 101107. DOI: 10.1016/j.mtphys.2023.101107.

(11) Bernges, T.; Hanus, R. C.; Wankmiller, B.; Imasato, K.; Lin, S.; Ghidiu, M.; Gerlitz, M.; Peterlechner, M.; Graham, S.; Hautier, G.; Pei, Y.; Hansen, M. R.; Wilde, G.; Snyder, G. J.; George, J.; Agne, M. T.; Zeier, W. G. Diffuson-mediated thermal and ionic transport in superionic conductors.

(12) Allen, P. B.; Feldman, J. L.; Fabian, J.; Wooten, F. Diffusons, locons and propagons: Character of atomic vibrations in amorphous Si. *Philos. Mag. B* **1999**, *79* (11-12), 1715–1731. DOI: 10.1080/13642819908223054.

(13) Hanus, R. C.; George, J.; Wood, M.; Bonkowski, A.; Cheng, Y.; Abernathy, D. L.; Manley, M. E.; Hautier, G.; Snyder, G. J.; Hermann, R. P. Uncovering design principles for amorphous-like heat conduction using two-channel lattice dynamics. *Mater. Today Phys.* **2021**, *18*, 100344. DOI: 10.1016/j.mtphys.2021.100344.

(14) Agne, M. T.; Hanus, R. C.; Snyder, G. J. Minimum thermal conductivity in the context of diffuson-mediated thermal transport. *Energy Environ. Sci.* **2018**, *11* (3), 609–616. DOI: 10.1039/C7EE03256K.

(15) Fallon, M. J.; Faka, V.; Lange, M. A.; Kraft, M. A.; Suard, E.; Connolly, E. T.; Francisco, B. E.; Squires, A. G.; Zeier, W. G. Exploring the Anion Site Disorder Kinetics in Lithium Argyrodites. *J. Am. Chem. Soc.* **2025**, *147* (12), 10151–10159. DOI: 10.1021/jacs.4c14466. Published Online: Mar. 12, 2025.
